# Supplementary figures and images for: Spatial subsidies drive sweet spots of tropical marine biomass production
Source: PLoS Biol. 2021 Nov 2;19(11):e3001435. doi: 10.1371/journal.pbio.3001435 (PMC8562822; doi:10.1371/journal.pbio.3001435)

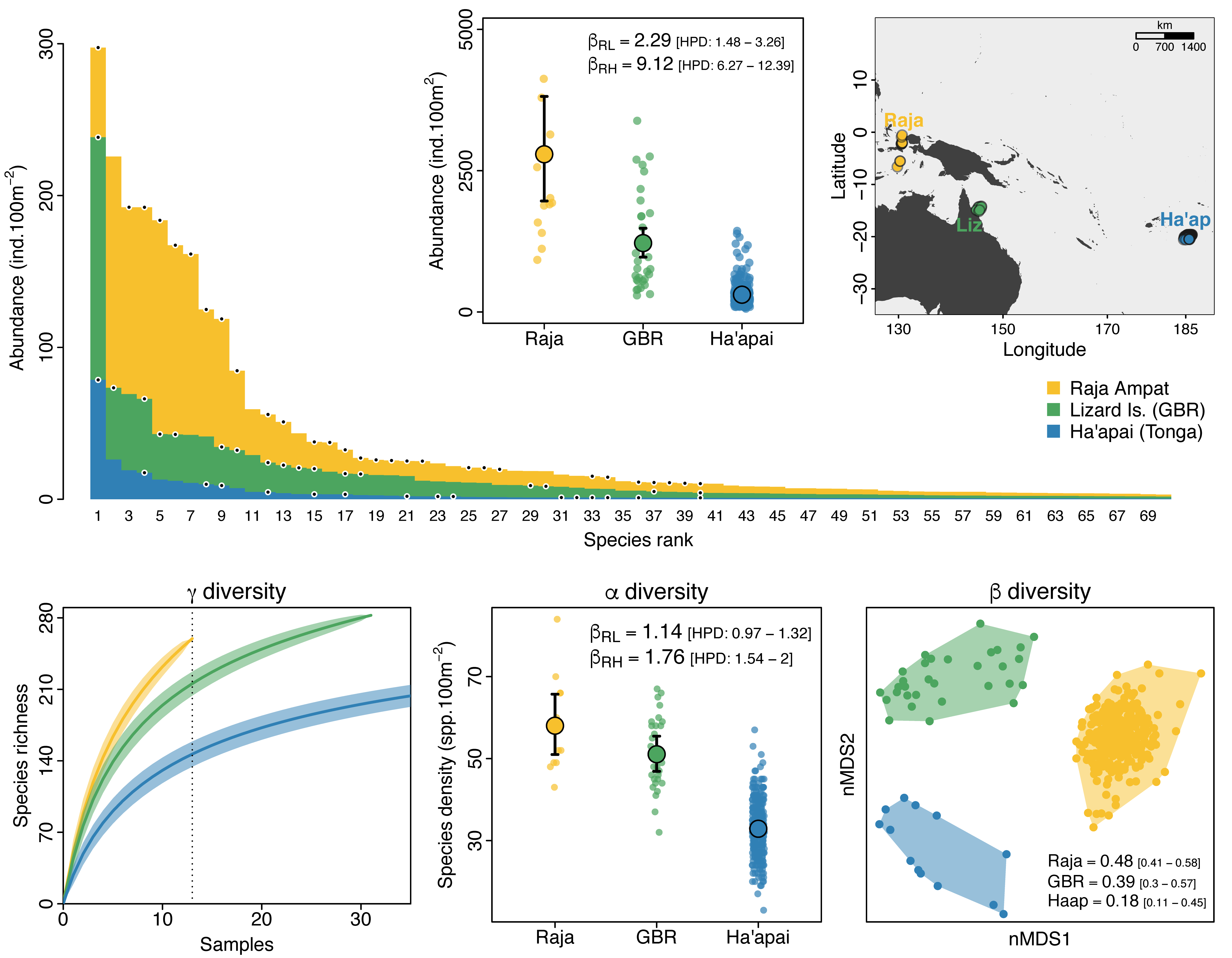

Supplement: S1 Fig — The top right inset shows a map with the sites sampled in each of the 3 locations. The species abundance relationship indicates only the 70 highest-ranking species at each location, with planktivores indicated by a black dot (for clarity, only planktivores among the 40 highest-ranking species displayed). Circles and intervals in the total fish abundance (top left inset) and α diversity (measured as sample-specific species density) represent Bayesian estimates and 95% HPD intervals, respectively. βRL is the ratio between model estimates in Raja Ampat and Lizard Island, and βRH between Raja Ampat and Ha’apai. Ribbons in the plot of γ diversity (measured from species rarefaction curves) are the 95% confidence intervals, while the dashed line represents the rarefaction point. The plot of β diversity (measured as the location-specific multivariate dispersion; see main text) shows the median and 95% quantile range of permutated multivariate dispersion. Map source: Natural Earth via the maps and mapdata packages in R. Numerical values underlying this figure are provided in “Morais_et_al_ FigS1.R,” available from https://doi.org/10.5281/zenodo.5540102. GBR, Great Barrier Reef; HPD, high posterior density. (TIF) [file pbio.3001435.s001.tif]

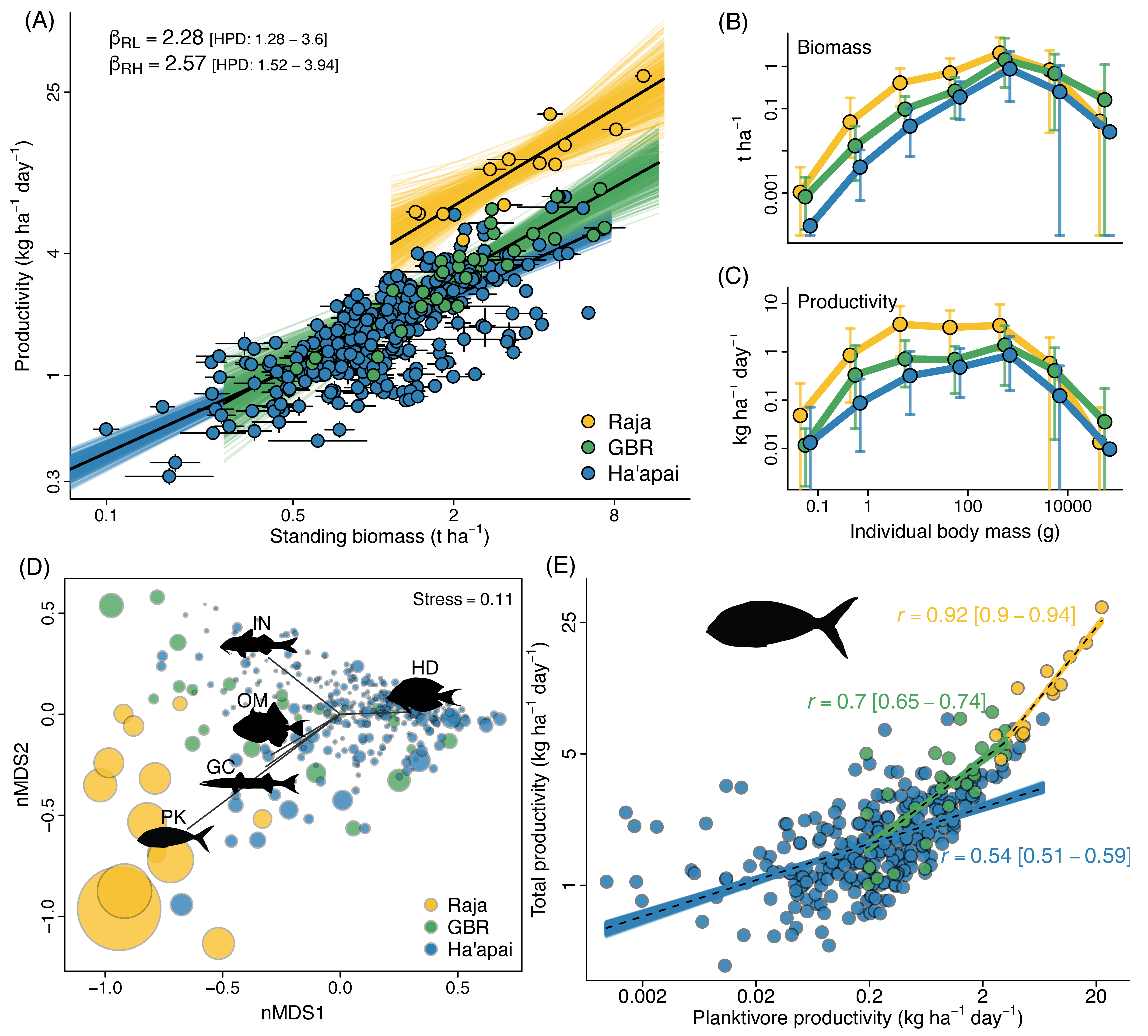

Supplement: S2 Fig — (A) Regression lines for all locations had a similar slope but different intercepts. Dots are the bootstrap medians for survey areas, and segments delimit the 95% bootstrap quantile intervals (see Methods). Coloured lines represent 500 draws from the Bayesian posterior distributions, and black lines represent the posterior medians. (B) Biomass and (C) productivity size distributions, with median (dots) and 90% bootstrap quantile intervals. (D) An nMDS biplot showing trophic groups as vectors and survey areas as circles scaled proportionally to total fish productivity. (E) Relationship between the productivity of planktivores and total fish productivity in the 3 locations. Trendlines depict 300 bootstrapped Pearson’s correlation r values (coloured lines) and median r (dashed lines) back calculated from standardised variables to productivity units (see Methods and S3 Fig) for each location. Circles are survey areas. Numerical values underlying this figure are provided in “Morais_et_al_Fig 03_FigS2.R,” available from https://doi.org/10.5281/zenodo.5540102. GBR, Great Barrier Reef; GC, generalised carnivores; HD, herbivores/detritivores; IN, invertivores; nMDS, nonmetric multidimensional scaling; OM, omnivores; PB, productivity–biomass; PK, planktivores. (TIF) [file pbio.3001435.s002.tif]

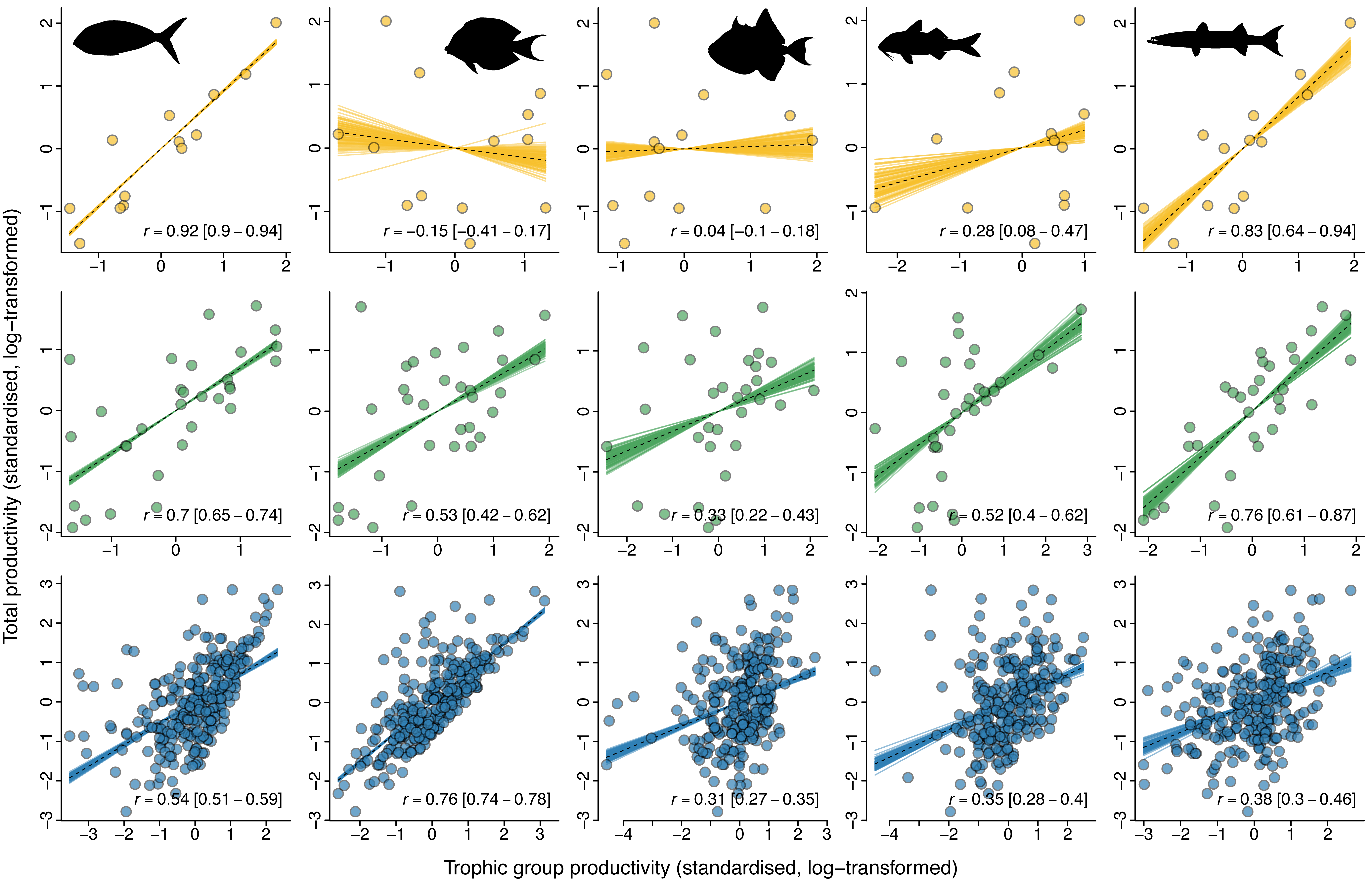

Supplement: S3 Fig — Raja Ampat: yellow, top row; Lizard Island: green, middle row; Ha’apai: blue, bottom row. Silhouettes denote, from left to right, planktivores, herbivores, omnivores, invertivores, and generalised carnivores. Trendlines depict 300 bootstrapped Pearson’s correlation r values (coloured lines) and median r (dashed lines). Labels provide the median and 95% quantile interval of r values for each trophic group, at each location. Notice the standardised, log-scale in the axes of the panels. Numerical values underlying this figure are provided in “Morais_et_al_FigS3.R,” available from https://doi.org/10.5281/zenodo.5540102. GBR, Great Barrier Reef. (TIF) [file pbio.3001435.s003.tif]

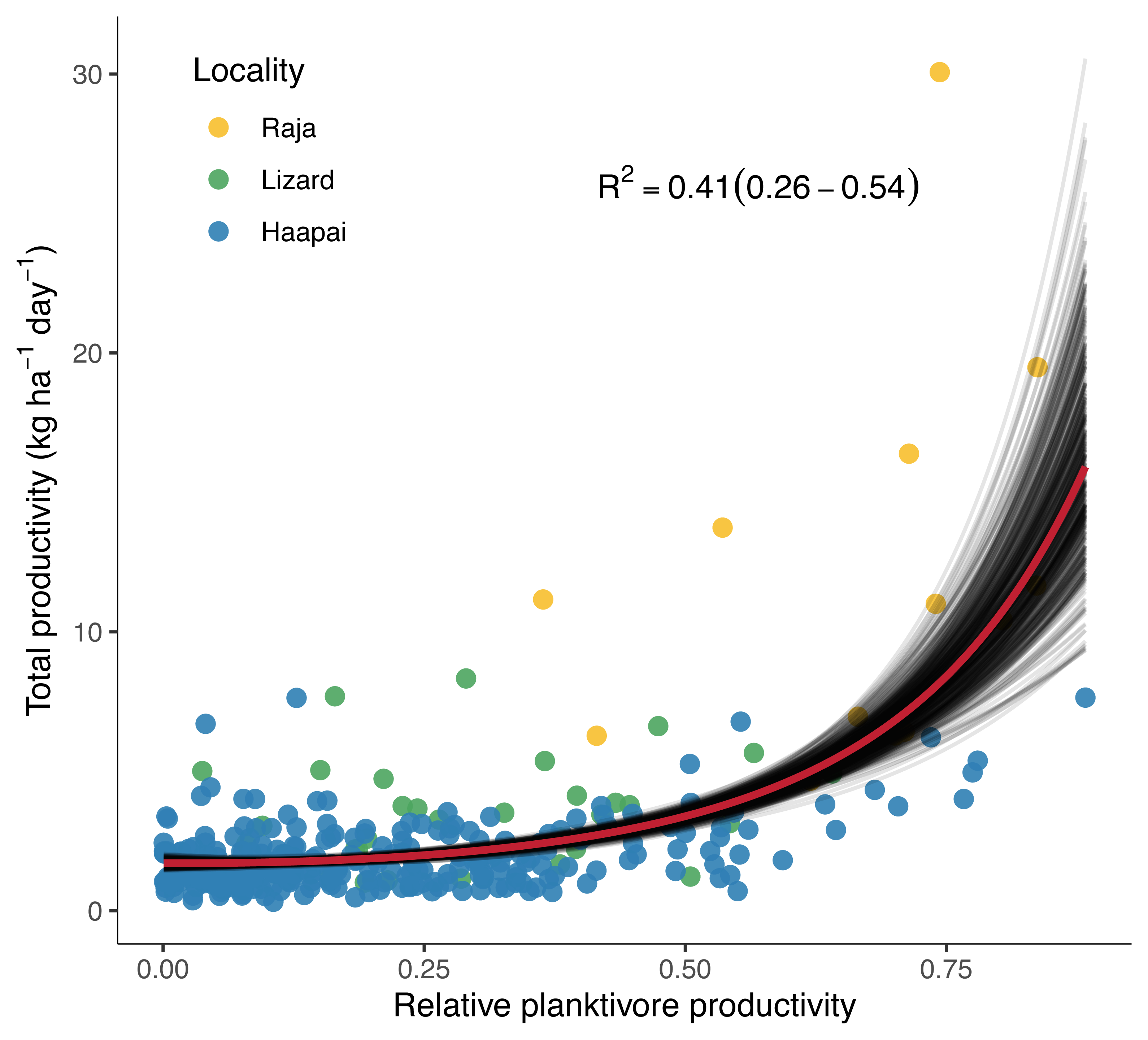

Supplement: S4 Fig — Circles are survey sites. Thin black lines are predictions from 500 draws, and the red line is the median prediction from the posterior distribution of the model coefficients. R2 values are the median and 95% high posterior distribution interval (in square brackets). Numerical values underlying this figure are provided in “Morais_et_al_FigS4.R,” available from https://doi.org/10.5281/zenodo.5540102. (TIF) [file pbio.3001435.s004.tif]

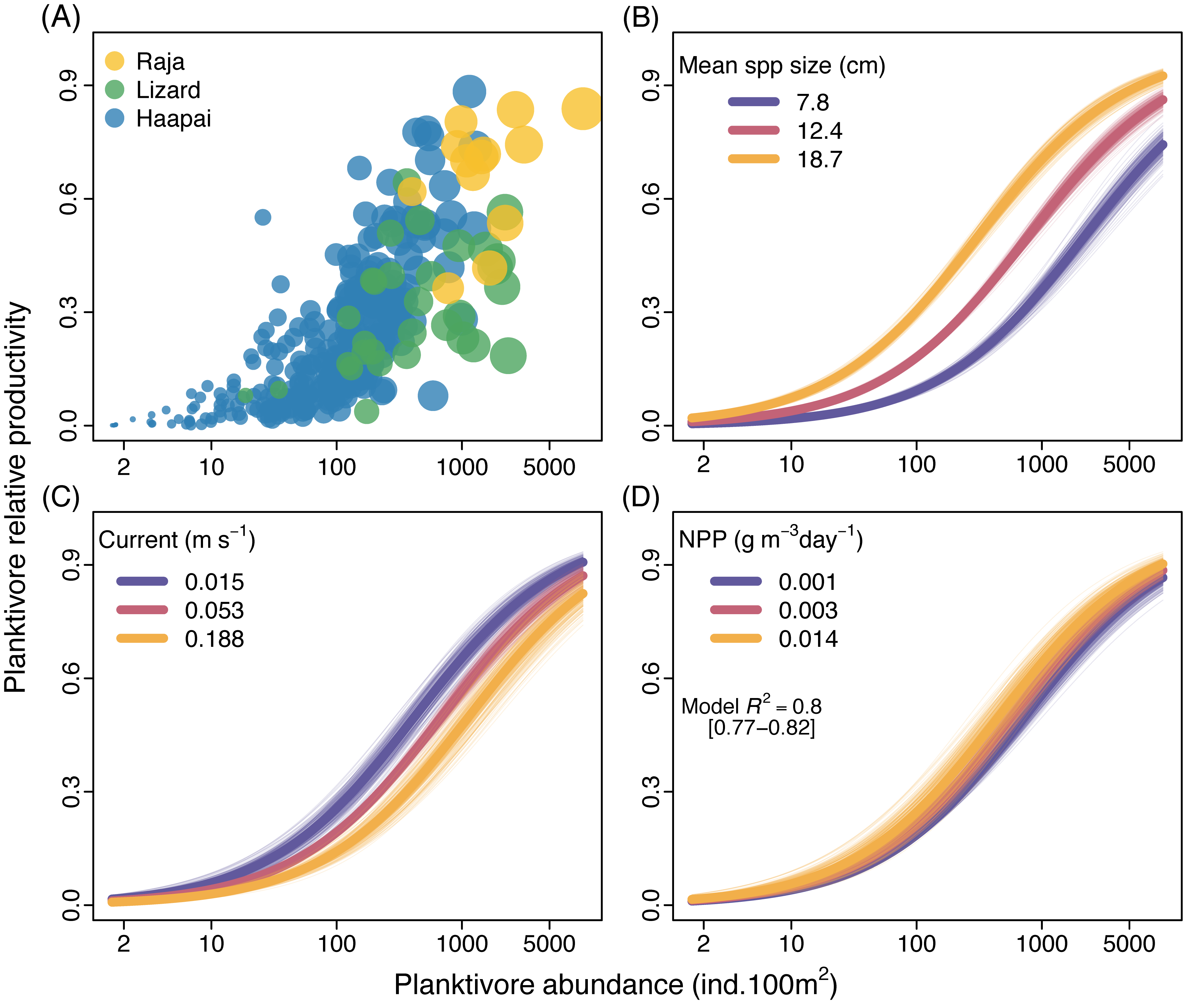

Supplement: S5 Fig — In (A), circles are proportional to the x-axis and represent survey sites. (B) Proportional planktivore productivity as a function of mean species size, (C) site-specific mean surface current velocity, and (D) and pelagic NPP. Thin lines are 500 draws from the posterior distribution of the coefficients describing the relationship, and thick lines describe the median predictions. Notice the log-scale in the x axis of the panels. Numerical values underlying this figure are provided in “Morais_et_al_FigS5.R,” available from https://doi.org/10.5281/zenodo.5540102. NPP, net primary productivity. (TIF) [file pbio.3001435.s005.tif]

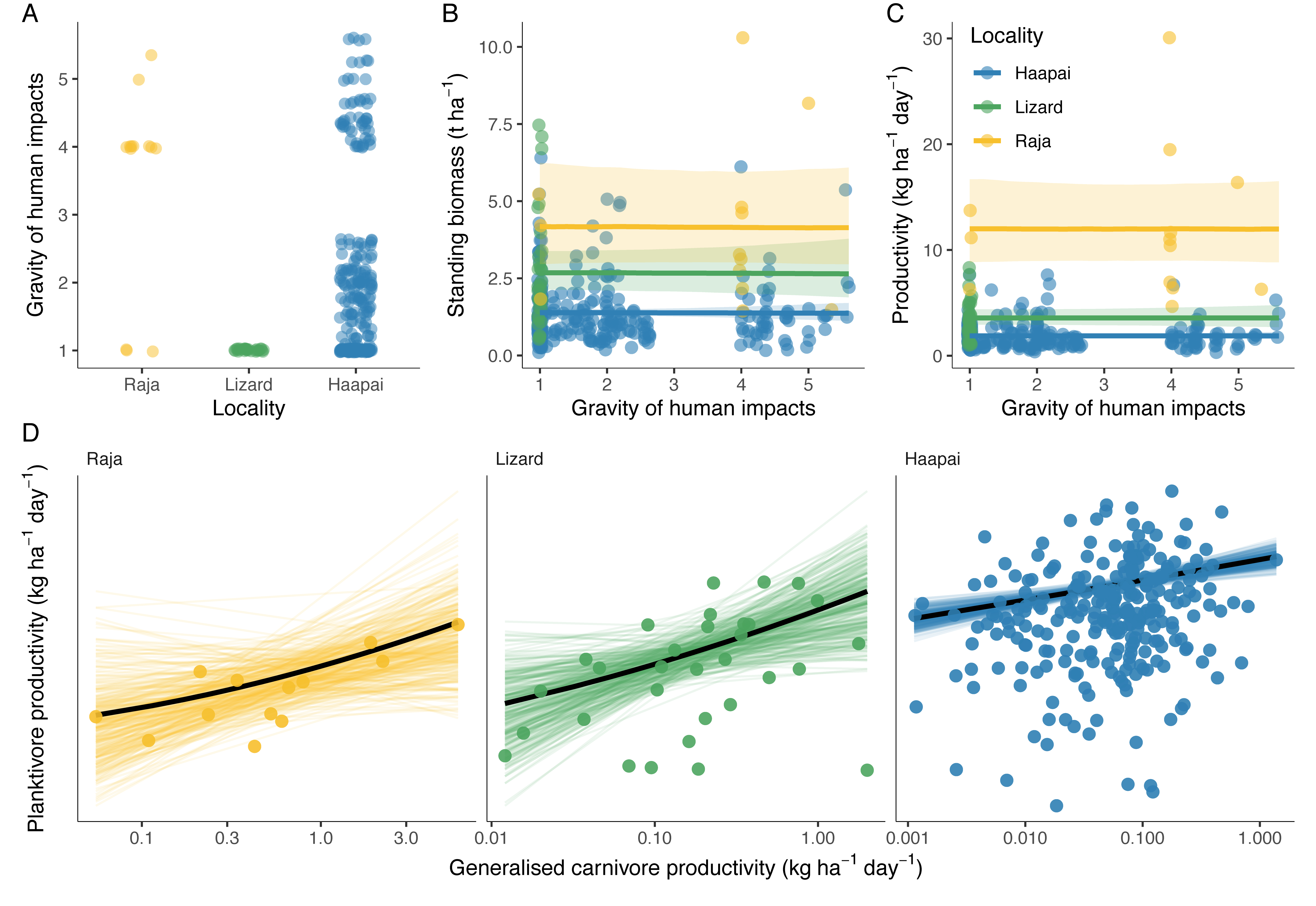

Supplement: S6 Fig — (A) The overall range of gravity of human impacts among sites in Raja Ampat and Ha’apai was similar but did not vary at Lizard Island, where it was minimal. (B) and (C) Modelling the potential effects of gravity on biomass (B) or productivity (C) showed a complete lack of relationship. Indeed, model selection procedures showed that a model including only locality performed better than any models including gravity. (D) The relationship between the productivity of generalised predators and the productivity of planktivores. This model tests for potential cascading effects from predator removal generating the trophic release of planktivores. Descending curves would provide evidence for this potential confounding process, yet the relationship was positive for the 3 localities. Thin lines are 400 draws from the posterior distribution of the coefficients describing the relationship, and thick lines describe the median predictions. Notice the log-scale in the x- and y-axis of the panels in (D). Numerical values underlying this figure are provided in “Morais_et_al_FigS6.R,” available from https://doi.org/10.5281/zenodo.5540102. (TIF) [file pbio.3001435.s006.tif]

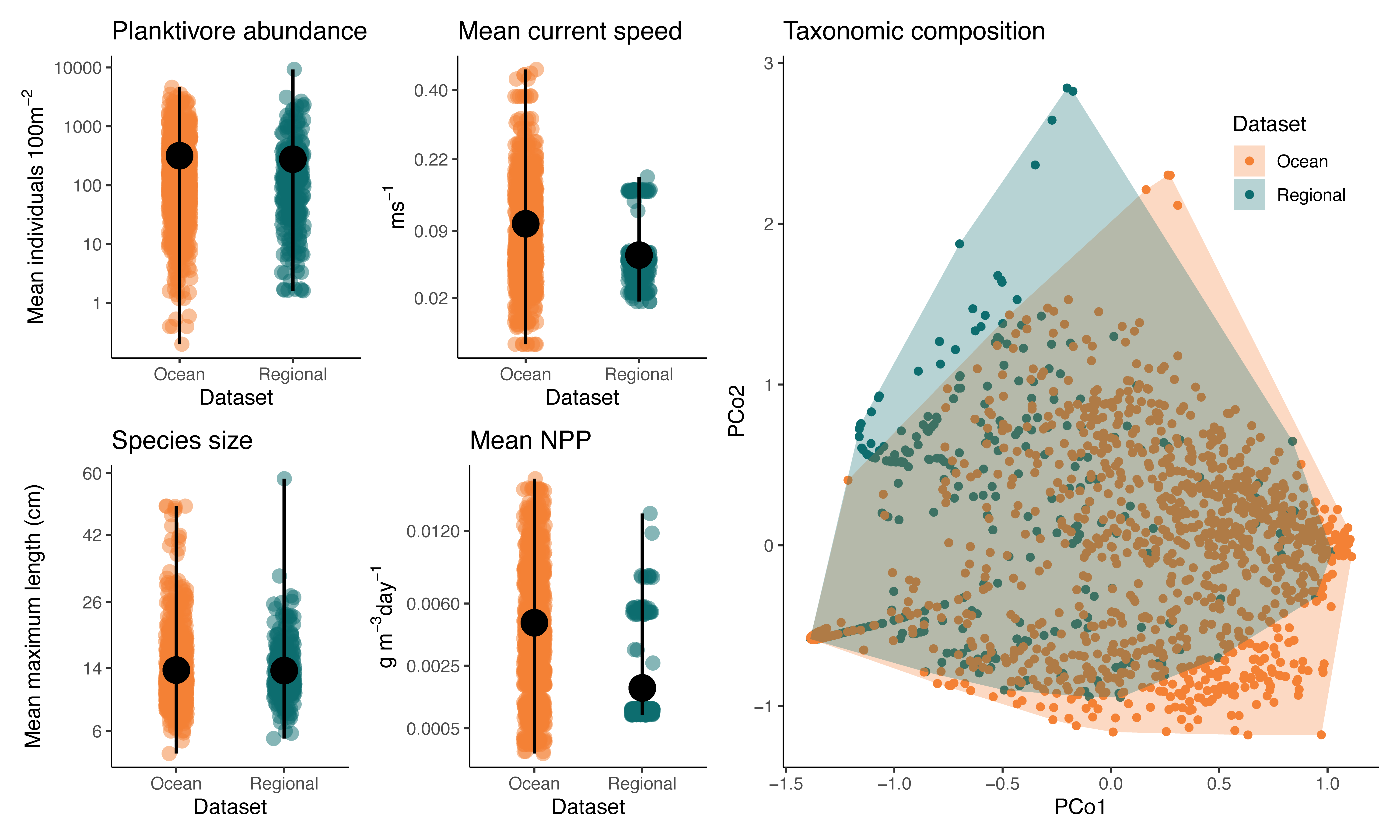

Supplement: S7 Fig — Coloured dots represent each site surveyed at each dataset, black circles represent mean values, and intervals represent the range. Notice that the y-axis is in the log-scale for planktivore abundance, and in the square root scale for mean current speed, species size, and mean pelagic NPP. These transformations were introduced to increase resolution of the lower values. The taxonomic composition ordination represents the 2 main axes of a PCoA, with polygons as the maximum convex polygon of each dataset. Numerical values underlying this figure are provided in “Morais_et_al_FigS7.R,” available from https://doi.org/10.5281/zenodo.5540102. NPP, net primary productivity; PCoA, principal coordinate analysis. (TIF) [file pbio.3001435.s007.tif]
