# Supplementary material for: Spatial subsidies drive sweet spots of tropical marine biomass production
Source: PLoS Biol. 2021 Nov 2;19(11):e3001435. doi: 10.1371/journal.pbio.3001435 (PMC8562822; doi:10.1371/journal.pbio.3001435)
Supplement: S1 Table — Estimates and variability are from the compounded chain including 1,000 Bayesian models run using Stan with the NUTS. Numerical values underlying this figure are provided in “Morais_et_al_Fig 03_FigS2.R,” available from https://doi.org/10.5281/zenodo.5540102. HPD, high posterior density interval (95%); NUTS, No-U-Turn Sampler. (DOCX) [file pbio.3001435.s008.docx]

**S1 Table |** Bayesian coefficients of the relationship between total reef fish standing biomass (predictor) and productivity (response) from Raja Ampat, Lizard Island and Ha’apai. HPD = high posterior density interval (95%). Estimates and variability are from the compounded chain including 1,000 Bayesian models run using Stan with the NUTS sampler. Numerical values underlying this figure are provided in ‘Morais_et_al_Fig03_FigS2.R’, available from https://doi.org/10.5281/zenodo.5540102.

| **Term** | **Coefficient** | **Std.Error** | **HPD.Low** | **HPD.High** |
| --- | --- | --- | --- | --- |
| Intercept | 1.44 | 0.23 | 0.99 | 1.91 |
| ln(Biom) | 0.76 | 0.17 | 0.42 | 1.09 |
| Locality - Lizard | -0.82 | 0.26 | -1.33 | -0.32 |
| Locality – Ha’apai | -0.95 | 0.24 | -1.41 | -0.49 |
| ln(Biom):Locality – Lizard | -0.03 | 0.20 | -0.42 | 0.36 |
| ln(Biom):Locality – Ha’apai | -0.16 | 0.17 | -0.50 | 0.18 |
