# Supplementary material for: Spatial subsidies drive sweet spots of tropical marine biomass production
Source: PLoS Biol. 2021 Nov 2;19(11):e3001435. doi: 10.1371/journal.pbio.3001435 (PMC8562822; doi:10.1371/journal.pbio.3001435)
Supplement: S2 Table — Estimates and variability are from the compounded chain including 1,000 Bayesian models run using Stan with the NUTS. Numerical values underlying this figure are provided in “Morais_et_al_Fig 04.R,” available from https://doi.org/10.5281/zenodo.5540102. HPD, high posterior density interval (95%); NPP, net primary productivity; NUTS, No-U-Turn Sampler. (DOCX) [file pbio.3001435.s009.docx]

**S2 Table |** Bayesian standardised coefficients of the relationship planktivorous fish abundance, maximum species size, mean surface current velocity and mean pelagic net primary productivity (predictors) and the proportional productivity of planktivorous fishes (response) from Raja Ampat, Lizard Island and Ha’apai. HPD = high posterior density interval (95%). Estimates and variability are from the compounded chain including 1,000 Bayesian models run using Stan with the NUTS sampler. NPP = net primary productivity. Numerical values underlying this figure are provided in ‘Morais_et_al_Fig04.R’, available from https://doi.org/10.5281/zenodo.5540102.

| **Term** | **Coefficient** | **Std.Error** | **HPD.Low** | **HPD.High** |
| --- | --- | --- | --- | --- |
| Intercept | -1.46 | 0.04 | -1.53 | -1.40 |
| log_10_(Planktivore abundance) | 1.13 | 0.05 | 1.04 | 1.21 |
| log_10_(Maximum species size) | 0.47 | 0.03 | 0.41 | 0.53 |
| log_10_(Mean surface current velocity) | -0.14 | 0.04 | -0.21 | -0.06 |
| log_10_(Mean Pelagic NPP) | 0.08 | 0.04 | 0.01 | 0.17 |
