# Supplementary material for: Spatial subsidies drive sweet spots of tropical marine biomass production
Source: PLoS Biol. 2021 Nov 2;19(11):e3001435. doi: 10.1371/journal.pbio.3001435 (PMC8562822; doi:10.1371/journal.pbio.3001435)
Supplement: S1 Text — (DOCX) [file pbio.3001435.s011.docx]

## Supplementary Text

### Does diversity drive abundance and productivity distinctions between Raja Ampat, GBR and Tonga?

Focusing on the three locations examined at the regional scale, Raja Ampat (Indonesia), Lizard Island (Great Barrier Reef) and Ha’apai (Tonga) revealed large differences in fish abundance. The surveys from Raja Ampat had on average 2.3 times higher fish abundance than the ones from Lizard Island, and over nine times higher than the ones from Ha’apai (**S1 Fig**). This observation begets the question: can these large differences in abundance, as well as the vastly distinct productivities (see main text, **Fig 2**), be driven simply by differences in species diversity between the three locations (*e.g.,* [42])? Whilst Indonesia spans the very core of the Indo-Australian Archipelago biodiversity hotspot, the Great Barrier Reef sits on a marginal position, and Tonga is located in the Central Pacific, over 6,000 km to the east, with reportedly lower diversity of corals and fish, for example [94,117–119]. Furthermore, under the biodiversity-ecosystem functioning hypothesis [120,121], mechanisms that allow for species coexistence and increased efficiency in resource exploitation (*i.e.,* such as niche complementarity and species packing [122–125]), are expected to translate differences in species diversity into comparable differences in ecosystem function. This may include, for instance, the energetic efficiency, use and productivity of biological communities [126,127]. Alternatively, gradients in abundance could be merely due to, for example, one, or a few exceptionally abundant dominant species.

The large differences in total fish abundance between Raja Ampat, Lizard Island and Ha’apai were not simply due to one, or a few, disproportionally abundant, dominant species. Indeed, the 40 highest-ranking species in Raja Ampat, in terms of abundance, were consistently more abundant than the 40 highest-ranking species in either Lizard Island or Ha’apai (**S1 Fig**). These differences in species abundance seemed to be largely driven by planktivorous-fish species. Indeed, planktivores represented 29 out of the 40 most abundant species in Raja Ampat, comprising 73% of individuals, but only 17 species comprising 56% of individuals at Lizard Island and 16 species comprising 45% of individuals in Ha’apai. Although Ha’apai had substantially lower γ and α diversity when compared to Raja Ampat (77 and 76% higher in Raja Ampat, respectively), and also lower β diversity (Ha’apai β_disper_ = 0.18 [95% quantile interval: 0.11 to 0.45]; Raja Ampat β_disper_ = 0.48 [95% quantile interval: 0.41 to 0.58]), the same was not true for Lizard Island. Indeed, the magnitude of difference between Raja Ampat and Lizard Island in γ and α diversities was much smaller than that for abundance (20 and 13% higher in Raja Ampat, respectively), while β diversity did not differ between the two locations (Lizard Island β_disper_ = 0.39 [95% quantile interval: 0.30 to 0.57]; **S1 Fig**). Thus, in our study, differences in detected species diversity were insufficient to explain the large disparity in abundance and also in productivity observed between the locations. Because these differences were also not simply due to one or a few dominant species, the most likely explanation is that there are system-wide energetic distinctions. Considering the relationships between the productivity of each trophic group and total fish productivity (**Fig 3E, S2 Fig, S3 Fig**) it appears parsimonious to conclude that these differences stem from planktivorous fishes and would not be predicted by differences in species diversity alone.

### Regional versus local drivers of high planktivore abundance and productivity

Our results also revealed a trend of increased planktivorous fish abundance, proportion, and productivity toward the ‘core’ of the Indo-Australian Archipelago. The core IAA encompasses Indonesia, Philippines, East Timor, Papua New Guinea and the Solomon Islands [93,94], largely matching the geographic boundaries of the ‘Coral Triangle’ [118,119]. This region has undergone intense tectonic activity for the past 45 million years and, as a result, hosts the maximum geological and geographical complexity in the tropics [128,129]. Largely because of this complex geological history, the IAA is regarded as the centre of marine biodiversity in the world [117,130–132], including higher species density compared to other regions (*i.e.,* 'species packing' [123,133]). Furthermore, the geographic and geomorphologic complexity of the core IAA underlie a peculiar oceanographic regime dominated by the powerful Indonesian throughflow (*i.e.,* the flow of Pacific water into the Indian Ocean [134,135]), but that also includes regional monsoon-driven upwelling and some of the fastest tropical tidal currents in the world [136,137]. Could the increased biodiversity, or the complex oceanographic features of the core IAA, be responsible for the spatial patterns detected? Although our focal locations span a large gradient in regional species richness, the extensive differences in total and planktivore productivity (mostly between Raja Ampat and the other two locations) were not mirrored by equivalent differences in species diversity (which occurred mostly between Ha’apai and the other locations). As an example, whilst total and planktivore productivity were, respectively, 230% and 630% higher in Raja Ampat compared to Lizard Island, local and regional diversity were only 13% and 20% higher in Raja Ampat than at Lizard Island. Oceanographic processes, on the other hand appear to contribute, at least to some extent, to planktivore productivity. This was evidenced by our model relating proportional productivity of planktivores to current speeds and pelagic primary productivity (**S5 Fig**). Nevertheless, while these variables were important to increase the predictive power of our model, their effect was smaller than planktivore abundance or species size, as evidenced by their smaller standardised coefficients (**S2 Table**). Overall, large-scale oceanographic variables appeared to be less important than local variability (see **S5 Fig**) in determining the ubiquitous role of planktivorous fishes in coral reef productivity.

The role of small-scale phenomena, such as local currents, is highlighted from the oceanic dataset, whereby most regions encompassed sites with both low and high planktivore abundance and relative productivity. Indeed, large-scale oceanographic principles have limited practical use in three-dimensional settings where the bottom topography strongly interacts with ocean currents and waves [58,138]. Instead, this interaction between currents and topography generates a complex array of secondary water flow phenomena that ultimately govern the transport of particles to, from, and through reefs [14,58,139,140]. In effect, coral reefs create their own oceanographic regimes [138] with flows like localised upwelling, longshore currents, island wake-generated eddies, and wave-driven transport, among others [14,39,58,140,141]. These localised water flows drive net plankton transport from the pelagic realm to reef consumers, underpinning the pelagic-benthic coupling and sustaining the so-called ‘wall of mouths’ of planktivorous fishes [39], but they are hard to predict from large-scale models or satellite-based oceanographic data.

### Spatial constraints of fish productivity on coral reefs

Our results showed that planktivores stand as the driving force behind exceptionally high fish productivity, composing 22.4 out of a maximum of 30 kg ha^-1^ day^-1^ produced from Raja Ampat. Although planktivore productivity in most survey areas were generally lower than this extreme value (with an average of ~8.2 kg ha^-1^ day^-1^), this was still considerably more than the maximum productivity observed for herbivorous fishes, for example (5.3 kg ha^-1^ day^-1^ in Ha’apai, 4.0 kg ha^-1^ day^-1^ at Lizard Island, average of 0.96 and 1.13 kg ha^-1^ day^-1^, respectively). These herbivore production rates are not different from the expected considering the usual benthic primary productivity of coral reefs. Algal turfs are the main benthic resource upon which herbivores rely, and are expected to contribute productivities between 0.4 and 1.4 g C m^-2^ day^-1^, when standardised by cover and rugosity (a similar range can be obtained from [142,143]). Assuming that 40-70% of this is consumed, ~50% of this being by herbivorous fishes (average turf production consumption rates from [144–146]), and a trophic transfer of 10-14% [5,147,148], this would result in maximum expected herbivorous fish production between 0.008 and 0.07 g C m^-2^ day^-1^. This range is in line with the maximum value observed in our data: 0.0134, 0.048 and 0.064 g C m^-2^ day^-1^, respectively in Raja Ampat, Lizard Island and Ha’apai (by considering 12% of 1.12, 4.00 and 5.3 kg ha^-1^ day^-1^ wet weight made by C [146] and converting mass and area units). Even values in the upper bound of reported turf productivities [149], 2.9 to 3.3 g C m^-2^ day^-1^, could only sustain herbivorous fish productivities of the order of 0.14-0.16 g C m^-2^ day^-1^ or, in wet weight units, ~11.7 to 13.3 kg ha^-1^ day^-1^. However, total fish productivity is thermodynamically constrained to be only a fraction of primary productivity. Thus, even if the potential productivity of higher trophic levels (*e.g.,* invertivores) is added, total fish productivity exclusively from internal photosynthesis would still fall far short of the maximum of 0.36 g C m^-2^ day^-1^ (from 30 kg ha^-1^ day^-1^ total wet weight) observed herein. Thus, internal primary productivity on coral reefs is bound to constrain the production of consumer biomass, unless it is circumvented by mobile consumers [2,17,19,21], or pelagic plankton subsidies [11,23,40,45].

Planktivores, indeed, may consume the productivity from a much larger area as zooplankton reaches reef fronts carried by water movements (*cf.* [16]). For example, the average planktivore productivity of 0.1 g C m^-2^ day^-1^ found in Raja Ampat (calculated from the 8.2 kg ha^-1^ day^-1^ as above) would require a net phytoplankton productivity of at least 100 times that, ~10 g C m^-2^ day^-1^. This assumes 10% of transfer efficiency between trophic levels, and that all zooplankton are herbivores. The first is a realistic assumption, but the second is very conservative [73,150]. Surface pelagic primary productivities ranging from 0.0026 to 0.0138 g C m-3 day^-1^ (average 0.0076 g C m^-3^ day^-1^), as obtained for coral reefs in the Raja Ampat region (from Bio-Oracle 2 [113], see Methods), would only be able to provide 0.0014-0.0073 g C m^-3^ day^-1^ given phytoplankton consumption rates of ~56% and conservative zooplankton consumption of 95% [151,152]. Hence, a catchment area between 1,370 and 7,143 m^2^ of surface pelagic waters [16] would be required to sustain each m^2^ of forereef occupied by planktivorous fishes in that region, every day. In practice, however, much of the zooplankton preyed upon reef fronts are carnivores (thus involving extra energy loss in the conversion), brought by productive subsurface currents rather than in oligotrophic surface waters [39,73,150]. Regardless of the magnitude of the phenomenon, these cursory calculations illustrate the fact that, in harvesting productivity from larger areas (transported to the reef by ocean currents), planktivorous fishes considerably expand the resource pool, and therefore the energetic and nutrient footprint, of coral reef consumers.

### Testing for potential effects of human exploitation

Overexploitation has been found to strongly shape the relationship between biomass and productivity, and thus the turnover of coral reef fish assemblages [43]. Could different levels of human exploitation among localities, or sites, in our regional dataset explain our results? Although we strived to exclusively incorporate sites that had limited or no fishing, we devised two additional analyses to address this question. These analyses comprised: 1) testing for potential effects of a proxy of human exploitation, the ‘gravity of human impacts’ from [101], on productivity and biomass (see Methods); and 2) testing for trophic release of planktivores from fishing-induced predatory fish depletion.

There was some variability in the gravity of human impacts among sites in two of our localities (Raja Ampat and Ha’apai, **S6A Fig.**). However, the maximum value across any of our sites was only 5.5, or around 38,000 times lower than the maximum human gravity found for coral reefs by [101]. This supports the hypothesis that none of our sites was exposed to intense fishing activities. Nevertheless, we performed four Bayesian Generalised Models, with biomass and productivity for each site as response variables (gamma distributed), and the following fixed effects: exclusively gravity (*Model 1*), exclusively locality (*Model 2*), an additive effect of gravity and locality (*Model 3*), and an additive effect of a spline function of gravity and locality (*Model 4*). These models comprised default priors, three chains, 3,000 iterations per chain, 50% burn-in and a thinning of one every two MCMC steps using the NUTS algorithm on Stan via *rstanarm* [89,90] in R. We compared these models using their expected log predictive density (ELPD) as calculated in the *loo* package in R [99]. We considered the model with the highest ELPD as the best model. For both biomass and productivity, all models including gravity were indistinguishable or performed worse (ELPD_Model1_ = -452.2, ELPD_Model3_ = -420.5, ELPD_Model4_ = -421.0) than the model that only included different intercepts for the different localities (ELPD_Model1_ = -419.1). Not surprisingly, including human gravity as one of the predictors of relative planktivore productivity in our predictive model (*i.e.,* from **Fig 4** and **S5 Fig**) also resulted in this variable being eliminated from the model due to lack of explanatory power (see Methods). Thus, we found no evidence of an effect of the gravity of human impacts on either fish biomass or productivity, between or within localities.

We also ran a Bayesian Generalised Linear Model with a gamma error structure and a log link to relate the productivity of predatory fishes (generalised carnivores, log-transformed predictor) and the productivity of planktivores (response). We used the same modelling framework and chain parameters as above. This aimed to test whether there was evidence of trophic release of planktivores due to the removal of predatory fishes by fishing that could be confounding some of our results. Support for this alternative hypothesis would come in the form of a negative relationship between the productivity of predatory fishes and planktivorous fishes. However, contrary to this expectation, we found a positive relationship between the productivity of predators and planktivores that was tighter for Raja Ampat, intermediate for Lizard Island, and noisier for Ha’apai – but that occurred in all three locations (**S6D Fig**). We interpret this as strong evidence for a lack of top-down effects from fishing on predatory and planktivorous fishes. Instead, this provides support for our overall hypothesis that intensified planktivore productivity (which we attribute to local scale oceanographic processes, see above *Regional versus local drivers of high planktivore abundance and productivity*) is passed on to their predators. As a result, places with high planktivore productivity also tend to have high predatory fish productivity.

### The expected contribution of planktivores to total fish productivity in the ocean-scale dataset

Extrapolating predictions of the contribution of planktivores to total fish production from a dataset spanning three selected locations to a near-global dataset could be problematic if predictors encompassed substantially smaller variability, or if the taxonomic composition was much more restricted in the regional dataset. To evaluate this possibility, we compared the density distributions of the four final predictors of the model between datasets: planktivore abundance, mean species size, mean surface current velocity and pelagic net primary productivity (NPP), as well as the family-level composition and structure. We found extensive overlap in the range of predictors from both datasets (**S7 Fig**), with most of the distinctions referring to extreme values that comprised only a small part of the total number of data points. The main disparities between datasets were on mean surface current velocities and pelagic NPP. In the first case, the highest current velocity values in the ocean dataset were not present in the regional dataset (**S7 Fig**), although mean values between datasets differed only 8.9% of the total range (Oceanic dataset = 0.100 m s^-1^; Regional dataset = 0.059 m s^-1^, combined range = 0.001 to 0.464 m s^-1^). In the case of pelagic NPP, although datasets had a larger overlap in their range of values, mean values were slightly more dissimilar: 18% (Oceanic dataset = 0.00475 g m^-3^ day^-1^; Regional dataset = 0.00162 g m^-3^ day^-1^, combined range = 0.000126 to 0.0176 g m^-3^ day^-1^).

The effect of these small distinctions in the distribution of predictor values between datasets on our model is not entirely clear, although there is evidence that they may be generating slight underestimates in the proportional productivity of planktivores for specific regions. To evaluate this, we contrasted predictions of our model at the ocean-scale with our regional level data by selecting sites in overlapping geographic areas. We did so by filtering surveys from the RLS dataset that were located within cells that included our sites from the regional scale dataset. These cells comprised 1.5° latitude and longitude at Lizard Island (Latitude = -15 to -13.5; Longitude = 144.5 to 146), 2° of latitude and longitude in Raja Ampat (Latitude = -2 to 0; Longitude = 129.5 to 131.5), 3° of latitude and longitude in Ha’apai (Latitude = -21 to -18; Longitude = -176.5 to -173.5).

For Raja Ampat, predictions of the relative productivity of planktivorous fishes in the ocean-scale dataset and estimated values from the regional dataset were very similar (Oceanic dataset mean = 0.48; range = 0.22 to 0.80; n = 37; Regional dataset mean = 0.67; range = 0.36 to 0.84; n = 13). For Lizard Island, 21 surveys from the oceanic dataset were located in the same geographic area, also indicating a similar mean contribution of planktivores to productivity (Oceanic dataset mean = 0.23; range = 0.06 to 0.66; n = 21; Regional dataset mean = 0.31; range = 0.04 to 0.64; n = 31). Finally, for Ha’apai, although mean contributions of planktivores to productivity were very similar between datasets, our model predicted a much narrower range of values (Oceanic dataset mean = 0.15; range = 0.01 to 0.32; n = 11; Regional dataset mean = 0.20; range = 0 to 0.88; n = 281). Remarkably, although 74 surveys from the regional dataset included planktivore relative productivity of over 30% (23 over 50%, up to a maximum of 88%), no sites from the oceanic dataset were predicted to have planktivore relative productivities over 32%. This re-emphasises the level of conservativeness of our model. Despite its high precision (Bayes R^2^ = 0.80), about 20% of the variance remained unexplained, likely due to unaccounted local-scale phenomena, which appear to drive ‘sweet spots’ of high planktivore productivity even in sites with unfavourable oceanographic conditions (*i.e.,* current speeds and pelagic NPP).

Finally, to estimate the overlap in the family-level taxonomic composition between the two datasets, we first calculated maximum convex polygons for each dataset based on the site coordinates from the ordination space (a Principal Coordinate Analysis, **S7 Fig**). Then, we estimated the area of each polygon, the union area of the polygons of the two datasets simultaneously, and the intersection between the polygons. All calculations were performed with spatial polygon tools using the R software packages *sp* and *rgeos* [153,154]. The area occupied by the two datasets was very similar: 5.53 ordination units^2^ (85.4% of the total) for the ocean-scale dataset and 5.46 ordination units^2^ (84.3% of the total) for the region-scale dataset. The overlapping area between polygons occupied 4.58 ordination units^2^, meaning 84.0% of the region-scale polygon, 82.9% of the ocean-scale dataset and 70.8% of the total polygon. This indicates that the taxonomic structures present in the two datasets were largely similar, particularly given that the ocean dataset comprised over three times as many sites as the region-scale dataset (1,028 vs. 325 sites).

## Supplementary References

117. Bellwood DR, Hughes TP, Connolly SR, Tanner J. Environmental and geometric constraints on Indo-Pacific coral reef biodiversity: Global coral reef biodiversity. Ecology Letters. 2005;8: 643–651. doi:10.1111/j.1461-0248.2005.00763.x

118. Allen GR. Conservation hotspots of biodiversity and endemism for Indo-Pacific coral reef fishes. Aquatic Conservation: Marine and Freshwater Ecosystems. 2008;18: 541–556. doi:10.1002/aqc.880

119. Veron JE, Devantier LM, Turak E, Green AL, Kininmonth S, Stafford-Smith M, et al. Delineating the Coral Triangle. Galaxea, Journal of Coral Reef Studies. 2009;11: 91–100.

120. Loreau M. Biodiversity and Ecosystem Functioning: Current Knowledge and Future Challenges. Science. 2001;294: 804–808. doi:10.1126/science.1064088

121. Hooper DU, Chapin FS, Ewel JJ, Hector A, Inchausti P, Lavorel S, et al. Effects of biodiversity on ecosystem functioning: a consensus of current knowledge. Ecological Monographs. 2005;75: 3–35. doi:10.1890/04-0922

122. Macarthur R, Levins R. The Limiting Similarity, Convergence, and Divergence of Coexisting Species. The American Naturalist. 1967;101: 377–385. doi:10.1086/282505

123. MacArthur R. Species packing and competitive equilibrium for many species. Theoretical Population Biology. 1970;1: 1–11. doi:10.1016/0040-5809(70)90039-0

124. Chesson P. Mechanisms of Maintenance of Species Diversity. Annual Review of Ecology and Systematics. 2000;31: 343–366. doi:10.1146/annurev.ecolsys.31.1.343

125. Levine JM, Bascompte J, Adler PB, Allesina S. Beyond pairwise mechanisms of species coexistence in complex communities. Nature. 2017;546: 56–64. doi:10.1038/nature22898

126. Brun P, Zimmermann NE, Graham CH, Lavergne S, Pellissier L, Münkemüller T, et al. The productivity-biodiversity relationship varies across diversity dimensions. Nature Communications. 2019;10: 5691. doi:10.1038/s41467-019-13678-1

127. Buzhdygan OY, Meyer ST, Weisser WW, Eisenhauer N, Ebeling A, Borrett SR, et al. Biodiversity increases multitrophic energy use efficiency, flow and storage in grasslands. Nature Ecology and Evolution. 2020;4: 393–405. doi:10.1038/s41559-020-1123-8

128. Hall R. Cenozoic geological and plate tectonic evolution of SE Asia and the SW Pacific: computer-based reconstructions, model and animations. Journal of Asian Earth Sciences. 2002;20: 353–431.

129. Lohman DJ, de Bruyn M, Page T, von Rintelen K, Hall R, Ng PKL, et al. Biogeography of the Indo-Australian Archipelago. Annual Review of Ecology, Evolution, and Systematics. 2011;42: 205–226. doi:10.1146/annurev-ecolsys-102710-145001130.

130. Connolly SR, Bellwood DR, Hughes TP. Indo-Pacific biodiversity of coral reefs: deviations from a mid-domain model. Ecology. 2003;84: 2178–2190. doi:10.1890/02-0254

131. Mora C, Chittaro PM, Sale PF, Kritzer JP, Ludsin SA. Patterns and processes in reef fish diversity. Nature. 2003;421: 933–936. doi:10.1038/nature01393

132. Tittensor DP, Mora C, Jetz W, Lotze HK, Ricard D, Berghe EV, et al. Global patterns and predictors of marine biodiversity across taxa. Nature. 2010;466: 1098–1101. doi:10.1038/nature09329

133. Barneche DR, Rezende EL, Parravicini V, Maire E, Edgar GJ, Stuart-Smith RD, et al. Body size, reef area and temperature predict global reef-fish species richness across spatial scales. Global Ecology and Biogeography. 2019;28: 315–327. doi:10.1111/geb.12851

134. Gordon AL. Oceanography of the Indonesian Seas and their Throughflow. Oceanography. 2005;18: 14–27.

135. Gordon AL, Fine RA. Pathways of water between the Pacific and Indian oceans in the Indonesian seas. Nature. 1996;379: 146–149. doi:10.1038/379146a0

136. Qu T, Du Y, Achan JS, Meyers G, Slingo J. Sea surface temperature and its variability in the Indonesian Region. Oceanography. 2005;18: 50–61.

137. Robertson R, Ffield A. M2 baroclinic tides in the Indonesian Seas. Oceanography. 2005;18: 62–73.

138. Hamner WM, Wolanski E. Hydodynamic forcing functions and biological processes on coral reefs. Proceedings of the 6th International Coral Reef Symposium. 1988. pp. 103–113.

139. Hamner WM, Hauri IR. Effects of island mass: Water flow and plankton pattern around a reef in the Great Barrier Reef lagoon, Australia. Limnology and Oceanography. 1981;26: 1084–1102. doi:10.4319/lo.1981.26.6.1084

140. Lowe RJ, Falter JL. Oceanic Forcing of Coral Reefs. Annual Review of Marine Science. 2015;7: 43–66. doi:10.1146/annurev-marine-010814-015834

141. Andrews JC, Gentien P. Upwelling as a Source of Nutrients for the Great Barrier Reef Ecosystems: A Solution to Darwin’s Question? Marine Ecology Progress Series. 1982;8: 257–269.

142. Russ GR. Grazer biomass correlates more strongly with production than with biomass of algal turfs on a coral reef. Coral Reefs. 2003;22: 63–67. doi:10.1007/s00338-003-0286-5

143. Klumpp D, McKinnon A. Community structure, biomass and productivity of epilithic algal communities on the Great Barrier Reef: dynamics at different spatial scales. Marine Ecology Progress Series. 1992;86: 77–89. doi:10.3354/meps086077

144. Carpenter RC. Partitioning Herbivory and Its Effects on Coral Reef Algal Communities. Ecological Monographs. 1986;56: 345–364. doi:10.2307/1942551

145. Polunin NVC, Klumpp DW. Algal food supply and grazer demand in a very productive coral-reef zone. Journal of Experimental Marine Biology and Ecology. 1992;164: 1–15. doi:10.1016/0022-0981(92)90132-T

146. Polunin NVC. Trophodynamics of reef fisheries productivity. In: Polunin NVC, Roberts CM, editors. Reef Fisheries. Dordrecht: Springer Netherlands; 1996. pp. 113–135. doi:10.1007/978-94-015-8779-2_5

147. Jennings S, Mackinson S. Abundance-body mass relationships in size-structured food webs. Ecology Letters. 2003;6: 971–974. doi:10.1046/j.1461-0248.2003.00529.x

148. Barneche DR, Allen AP. The energetics of fish growth and how it constrains food-web trophic structure. Ecology Letters. 2018;21: 836–844. doi:10.1111/ele.12947

149. Carpenter RC. Relationships between primary production and ii radiance in coral reef algal communities. Limnology and Oceanography. 1985;30: 784–793. doi:10.4319/lo.1985.30.4.0784

150. Hobson ES. Trophic Relationships of Fishes Specialized to Feed on Zooplankters above Coral Reefs. In: Sale PF, editor. The Ecology of Fishes on Coral Reefs. Elsevier; 1991. pp. 69–95. doi:10.1016/B978-0-08-092551-6.50009-X

151. Arias-Gonzalez JE, Delesalle B, Salvat B, Galzin R. Trophic functioning of the Tiahura reef sector, Moorea Island, French Polynesia. Coral Reefs. 1997;16: 231–246. doi:10.1007/s003380050079

152. Bozec Y-M, Gascuel D, Kulbicki M. Trophic model of lagoonal communities in a large open atoll (Uvea, Loyalty islands, New Caledonia). Aquatic Living Resources. 2004;17: 151–162. doi:10.1051/alr:2004024

153. Bivand RS, Pebesma E, Gomez-Rubio V. Applied spatial data analysis with R. Second edition. Springer, NY; 2013. Available: https://asdar-book.org/

154. Bivand R, Rundel C. rgeos: Interface to Geometry Engine - Open Source ('GEOS’). 2019. R package version 0.5-2. Available: https://CRAN.R-project.org/package=rgeos
