# Supplementary material for: Spatial subsidies drive sweet spots of tropical marine biomass production
Source: PLoS Biol. 2021 Nov 2;19(11):e3001435. doi: 10.1371/journal.pbio.3001435 (PMC8562822; doi:10.1371/journal.pbio.3001435)
Supplement: Attachment — Submitted filename: Morais et al. Responses to Reviewers.docx [file pbio.3001435.s012.docx]

**REVIEWERS' COMMENTS:
Reviewer #1:
[identifies himself as James Robinson]**

Morais et al. use a large underwater census dataset to show that pelagic (planktonic) subsidies are a major contributor to coral reef productivity, and these subsidies enable some reef systems to have exceptionally high levels of local biomass production. This study has the potential to considerably advance our understanding of biomass production on reefs by demonstrates a novel empirical link between biomass hotspots and pelagic energy inputs. The study question builds on theory predicting the importance of energetic subsidies to reef systems. The dataset is impressive, and well-suited to answer this question. The manuscript is well-written with thoughtful and impactful figures.

I believe the overall study question, data and context can be key advances, and should be suitable for publication in PLoS Biology. However, there are several fundamental problems in the Bayesian approaches used here. The method issues need addressed and the results may change. 

**Authors:** Thank you for your careful review and positive comments. As you will note from the responses below, both our Bayesian bootstrap approach and the use of ratios for comparison between localities are statistically and conceptually sound, and therefore easily justifiable. We believe your concerns may have arisen from a paucity of details explaining these methods, which we have amended in both instances. As for your third point, we agree that regression analyses are not the ideal approach in these cases due to the assumption of independence between variables, but also because implying causal relationships in these cases were not our intent. Therefore, we have replaced the regressions with correlations. We provide detailed responses to each of your comments below. Once more, thank you very much for offering the time to review our manuscript, helping to streamline and clarify some of the methods employed.

First, I don't understand the justification for bootstrapping Bayesian models, as this seems to introduce potential problems with model convergence that cannot be inspected. For example, you cannot assess the Rhat, effective samples and convergence of each 1000 models (1 per bootstrap sample), and so cannot be confident that the compounded posterior contains 100% valid samples. I see no reason why you wouldn't fit one Bayesian model to each response metric, as you are already calculating the mean response metric across iterations (L311). (it is also advisable to use the median of a bootstrap sample as this is robust to outliers, rather than the mean).

I would suggest re-running each analysis on the median bootstrapped ecological value, thoroughly inspecting model diagnostics, and using those model posteriors for inference. Or if there is serious justification for your approach (I cannot see it, and you do not cite these approaches), then adding material that summarizes the R-hat and convergence estimates for all 1000 models is necessary. But fitting one model per metric would be safer, just as robust (likely more), easier to implement and clearer to understand.  

**Response 01:** The bootstrap method used was designed to propagate uncertainty in the community metrics estimated (i.e., biomass and productivity) over to the final analyses. Propagating error and uncertainty is fundamentally aligned with the Bayesian approach. Although this approach is still not commonly used ecological studies, we believe it is an excellent way of dealing with data like ours that arises from resampled distributions. In these cases, using a simple median from resampled distributions may not be enough to incorporate all the variability in the metrics quantified. Your comments seem to be based in two arguments, which we address below.

First, that inspecting the MCMC diagnostics for all chains of all models would be hard or impossible. This important procedure is trivial and had been embedded in the code from the start, in the form of automated logical checks performed for each one of the 1,000 models, for each analysis. These automated logical checks were based on the effective sample sizes (a minimum of 100 times the number of chains for each parameter) and Rhat values (all below 1.05), as the reviewer correctly pointed out. Whenever a model was judged against these criteria, and failed, that model was excluded from the final, compound set of MCMC chains. These check have been summarised and are now listed in the methods, for each modelling procedure, citing the number of models (from a total of 1,000 models per procedure) that passed each of the criteria above. Only these models were retained for the final posterior distributions, giving us full confidence on the convergence of all Bayesian procedures employed.

Second, the suggestion that reruning these analyses using the median values of the community metrics analysed, instead of the bootstrapping approach, would be superior. We have now performed this and provide below a table comparing estimates and the high posterior density intervals, for all modelling procedures, between these two contrasting strategies. While differences between the two approaches depended on the model and parameter being estimated, overall, the bootstrap approach resulted in broader high posterior density intervals (see below), indicating it propagates more uncertainty than simply using resample medians. Therefore, this approach is more conservative. At the same time, estimates of parameters (i.e., posterior medians) were similar, showing that the power of the analysis remains unchanged. Although in our case the differences were often not of large magnitude, this will not necessarily always be the case. In cases where there is larger variability in the outputs from resampled datasets, we expect the bootstrap approach to be significantly more conservative than your suggestion. Because we believe that this is the most philosophically sound approach, we stand by our choice of bootstrapping the Bayesian models and keep this procedure throughout the analyses. We have revised our description of these procedures in the methods, adding more details and organising it in its own section ‘*Statistical modelling procedures*’ (see lines 414-438). We hope that this will clarify the reasoning for bootstrapping Bayesian models, as we thoroughly believe that it constitutes a robust way of benefitting from the power of Bayesian analyses while simultaneously propagating uncertainty and variability.

**Table 1:** Comparison of parameter estimates (Coefficient), standard errors and high posterior density intervals between two approaches: 1,000 models, each run with bootstrapped variables from resampling distributions (Bootstrap); and only one model run with resampled medians (Median).

| **Model** | **Term** | **Approach** | **Coefficient** | **Std.Error** | **HPD.Low** | **HPD.High** |
| --- | --- | --- | --- | --- | --- | --- |
| Abundance | Ha’apai | Bootstrap | 807.240 | 289.220 | 327.350 | 1277.920 |
|  |  | Median | 806.150 | 14.070 | 777.890 | 832.190 |
|  | Lizard | Bootstrap | 1722.020 | 302.350 | 1185.030 | 2247.950 |
|  |  | Median | 1713.200 | 92.470 | 1551.480 | 1904.130 |
|  | Raja | Bootstrap | 3319.300 | 405.810 | 2481.270 | 4046.690 |
|  |  | Median | 3269.530 | 264.310 | 2814.940 | 3836.400 |
|  | RatioRH | Bootstrap | 4.060 | 1.700 | 2.600 | 8.210 |
|  |  | Median | 4.060 | 0.330 | 3.480 | 4.790 |
|  | RatioRL | Bootstrap | 1.890 | 0.290 | 1.480 | 2.550 |
|  |  | Median | 1.910 | 0.180 | 1.560 | 2.270 |
| Spec density | Ha’apai | Bootstrap | 32.880 | 0.510 | 31.870 | 33.890 |
|  |  | Median | 32.950 | 0.500 | 31.950 | 33.910 |
|  | Lizard | Bootstrap | 51.080 | 2.190 | 46.880 | 55.460 |
|  |  | Median | 51.130 | 2.120 | 47.130 | 55.240 |
|  | Raja | Bootstrap | 57.970 | 3.760 | 50.840 | 65.530 |
|  |  | Median | 57.880 | 3.600 | 51.510 | 65.570 |
|  | RatioRH | Bootstrap | 1.760 | 0.120 | 1.540 | 2.000 |
|  |  | Median | 1.760 | 0.110 | 1.550 | 1.990 |
|  | RatioRL | Bootstrap | 1.130 | 0.090 | 0.970 | 1.310 |
|  |  | Median | 1.130 | 0.080 | 0.960 | 1.290 |
| Biomass | Ha’apai | Bootstrap | 1.387 | 0.056 | 1.279 | 1.501 |
|  |  | Median | 1.385 | 0.054 | 1.278 | 1.486 |
|  | Lizard | Bootstrap | 2.675 | 0.343 | 2.066 | 3.392 |
|  |  | Median | 2.664 | 0.326 | 2.081 | 3.340 |
|  | Raja | Bootstrap | 4.146 | 0.825 | 2.769 | 5.891 |
|  |  | Median | 4.097 | 0.791 | 2.824 | 5.774 |
|  | RatRH | Bootstrap | 2.988 | 0.609 | 1.984 | 4.287 |
|  |  | Median | 2.967 | 0.585 | 2.023 | 4.186 |
|  | RatRL | Bootstrap | 1.549 | 0.371 | 0.937 | 2.336 |
|  |  | Median | 1.553 | 0.359 | 0.961 | 2.375 |
| Productivity | Ha’apai | Bootstrap | 1.885 | 0.062 | 1.767 | 2.010 |
|  |  | Median | 1.888 | 0.062 | 1.762 | 2.005 |
|  | Lizard | Bootstrap | 3.580 | 0.364 | 2.918 | 4.329 |
|  |  | Median | 3.571 | 0.356 | 2.940 | 4.313 |
|  | Raja | Bootstrap | 11.947 | 1.901 | 8.693 | 15.962 |
|  |  | Median | 11.904 | 1.860 | 8.470 | 15.624 |
|  | RatRH | Bootstrap | 6.337 | 1.031 | 4.567 | 8.513 |
|  |  | Median | 6.291 | 1.018 | 4.480 | 8.346 |
|  | RatRL | Bootstrap | 3.337 | 0.634 | 2.251 | 4.674 |
|  |  | Median | 3.324 | 0.611 | 2.184 | 4.531 |
| Prod vs Biom | IntHaap | Bootstrap | 0.494 | 0.022 | 0.451 | 0.538 |
|  |  | Median | 0.494 | 0.021 | 0.452 | 0.535 |
|  | IntLiz | Bootstrap | 0.617 | 0.104 | 0.412 | 0.822 |
|  |  | Median | 0.583 | 0.101 | 0.389 | 0.787 |
|  | IntRaja | Bootstrap | 1.439 | 0.234 | 0.983 | 1.902 |
|  |  | Median | 1.424 | 0.225 | 1.003 | 1.876 |
|  | IntRatRH | Bootstrap | 2.910 | 0.494 | 1.964 | 3.899 |
|  |  | Median | 2.886 | 0.474 | 1.996 | 3.828 |
|  | IntRatRL | Bootstrap | 2.335 | 0.602 | 1.341 | 3.604 |
|  |  | Median | 2.457 | 0.612 | 1.510 | 3.839 |
|  | SlopHaap | Bootstrap | 0.596 | 0.032 | 0.534 | 0.658 |
|  |  | Median | 0.605 | 0.030 | 0.550 | 0.667 |
|  | SlopLiz | Bootstrap | 0.729 | 0.105 | 0.526 | 0.937 |
|  |  | Median | 0.766 | 0.103 | 0.576 | 0.970 |
|  | SlopRaja | Bootstrap | 0.758 | 0.170 | 0.426 | 1.093 |
|  |  | Median | 0.770 | 0.164 | 0.449 | 1.093 |
|  | SloRatRH | Bootstrap | 1.271 | 0.294 | 0.703 | 1.858 |
|  |  | Median | 1.271 | 0.280 | 0.749 | 1.858 |
|  | SloRatRL | Bootstrap | 1.038 | 0.290 | 0.521 | 1.646 |
|  |  | Median | 1.003 | 0.264 | 0.505 | 1.532 |

Second, you use the ratio of posterior distributions to infer differences in site-level intercepts. Differences between Bayesian posteriors can be assessed by summation or differencing (Hobbs & Hooten, Bayesian Models, pg. 195), which would provide a confidence interval or Bayesian p-value for the difference between locations (in this case). For example, in Fig. 2, the ratio of location-intercept posteriors does not show that there are different intercepts. Rather, you would infer differences by comparing the posteriors of location-intercepts (do intervals overlap?), or by estimating the difference between posteriors of location-intercepts. I don't follow that ratios of distributions allows you to estimate certainty intervals either, though you use these statistics throughout the results (L103-108).

**Response 02:** MCMC sampling provides the flexibility to marginalise parameters independently at each iteration and to perform calculations with derived quantities exactly the same way one would do with the parameter estimates themselves. This is explained, for example, in the same book you quoted (pg. 194): “*The idea of equivariance is that any quantity that is calculated from a random variable becomes a random variable with its own probability distribution*.” and also “*The equivariance property of MCMC allows us to easily obtain posterior distributions of these derived quantities—a difficult if not impossible task using classical statistics.*”.

The ratios we calculate here follow the same logic of the differences you suggest above. The main distinction between these two approaches is that, while differences range from negative to positive values (in units of the variable), ratios operate on a 0 < Ratio < Infinite interval (with relative units). Regardless, their interpretation is analogous: the closer the ratio is to 1 (and a difference to 0), and the more the area of its 95% credibility interval overlaps 1 (or 0 for differences), the lower the probability of differences in the coefficients compared. However, ratios allow proportional differences to be spotted straight away. A ratio of 1.6 between two coefficients, for example, implies that the first coefficient exceeds the second by 60%. Calculating high posterior density intervals for these derived quantities is, as stated by Hobbs and Hooten above, a fundamental property of MCMC sampling. A convenient way of calculating both differences and ratios is the emmeans package in R, which will return differences if contrasts are made at the link scale, and ratios if contrasts are made at the response scale (we do, however, use custom code for a similar result).

As you mentioned, reporting ratios does not immediately provides access to the values of the coefficients estimated (median of the posterior distribution). However, whenever we felt that was needed, we have provided the values of the coefficients (*e.g.,* lines 107-113). Regarding the specific example you cited, we have now more clearly pointed to Table S1, where the values of the intercepts can be accessed, and also expanded this sentence by listing all coefficients [please, note that some of the coefficients have suffered minor numerical changes as another round of 1,000 bootstrapped analyses were run]. It now reads (lines 113-118):

“The three locations had a similar rate of increase in productivity per unit biomass (i.e., slope, Figure 3A), yet there were substantial differences in their basal level of productivity (i.e., intercept; Figure 3A, Table S1). For the same values of biomass, fish productivities were 2.3 times larger in Raja Ampat compared to Lizard Island (βpbr = 2.34, HPD = 1.34 to 3.60; Fig. 3A, Fig. S2) and almost three times larger than in Ha’apai (βpbr = 2.91, HPD = 1.96 to 3.90) (for coefficient values, see Table S1).”

Third, in two analyses you fit a regression to the equation trophic abundance ~ total abundance, and total productivity ~ planktivore productivity. In this model structure, Y (e.g. total abundance/prod.) is directly dependent on X (e.g. planktivore abundance/prod.), violating model assumptions. As in your other analyses, why not fit relative planktivore abundance/prod. ~ total abundance/prod.? This affects Figs. 3, S3, S4.

**Response 03:** This is a good point, thanks for raising it. Although we did not fit a regression of the form you specified for abundance (it had, instead, relative trophic abundance ~ total abundance), we indeed fitted such a regression for productivity. The goal of this analysis has never been to infer causality, as we agree regressions imply. Rather, the goal has been to simply quantify correlations. As you are aware, correlations can be quantifed from the slope of regressions when variables are standardised. However, that is not how the results were reported, as we only reported the slopes of the log regressions. Therefore we recognise that the regressions resulted in violation of the assumption of independence, although this was unwanted and unecessary.

To amend this conceptual slip, following the reviewer’s advice, we have now re-analysed all five models of trophic productivity versus total productivity using standardised log-variables while also fixing intercepts to 0. We now report correlation coefficients for each trophic group, separately for each locality, in Fig. S3. In Fig. 3E, we have back-calculated trendlines from the standardised (z-score) values, and thus present a single plot at the productivity scale – instead of a z-score scale. Methods, and all results mentions have been changed accordingly (lines 495-505, Figs. 3E and S3; former Fig. S4 has been excluded and is now incorporated in Fig. S3). Thank you again for bringing this issue to our attention.

I realise these recommendations are significant and apply to most of your statistical analysis. I must stress that I expect the overall result - planktivores contributing to most reef fish productivity, and predicting reef sweet spots - will be robust. But I see little justification for bootstrapping Bayesian models (have you an example of someone doing this?). Taking posterior distribution ratios seems mathematically incorrect. It is impossible to assess how many invalid posterior samples occur in these compounded posterior distributions.

**Response 04:** Indeed, where analyses have been modified (i.e., correlations instead of regressions), results have remained unchanged. Please, refer to **Response 01** above for clear explanations of the bootstrapping procedure as a means of propagating uncertainty while performing robust estimation of parameters. As stated there, examiming the convergence of the boostrapped Bayesian models is a trivial matter. Please, also refer to **Response 02**, where we expose the mathematical soundness and the statistical validity of calculating derived metrics using posterior distributions.

Minor comments

L44 - study [23] did not find any correlation between primary productivity and fish biomass, but hypothesized that high biomass = subsidy effect. There is an important distinction here between planktonic subsidies to planktivores, and pelagic ecosystem subsidies to reef piscivores (Doug McCauley's papers). This distinction should be clearer throughout - that you are testing for planktonic energy input to planktivorous fishes, though other pelagic subsidies are also known to promote biomass of upper trophic levels through different pathways (mobile consumers, foraging etc.).

**Response 05:** We have now clarified that study [23] only hinted to this link by including the word ‘potential’ before ‘direct link’ (line 43), and also specified which type of subsidy we were talking about (“i.e., plankton”). We do agree that there is a distinction between these direct and indirect pathways through which subsidies reach reef food webs. Although we don’t think there is sufficient evidence to be specific at this point, we have now included a sentence clarifying this in the last paragraph of the Introduction. This now reads (lines 67-69):

“We focus solely on plankton subsidies incorporated by planktivorous fishes. We do not consider indirect trophic links that may also result in these subsidies being incorporated by reef communities (e.g., predators of planktivorous fishes, see Methods).”

Figure 3E and 4B - I find that scaling point size by productivity unhelpful as it hides data. Colour is sufficient here, and we would better understand the number and distribution of sweetspots if points are the same size. E.g. panel C shows many more sites than B, but is this just because the large points in B obscure many sites? Same applies to Fig 3E, as many points appear to cluster together.

**Response 06:** We agree with the reviewer that sometimes this is unnecessary. Because of that, we have removed scaling of point sizes in Fig. S3 (which now includes former Figs. S3 and S4) and in Fig. S4 (former Fig. S5). However, we also believe that scaling dots by a third variable is a useful resource for Figs. 3E and 4B. Although we have decided to keep the scaling in these cases, we made changes to avoid the issues you raised. In the case of Fig. 3E, we now provide the same plot, with all data points unscaled, as Fig. S2. In the case of Fig. 4B, we believe that a correct interpretation could have been hindered, as you mentioned, by a lack of information on the number of sites in each category (‘sweet spots’, 4B; of ‘low planktivore productivity spots’, 4C). Therefore, we now explicitly state the number of sites in the title of each panel. Thank you for raising this point.

L150 - this is a great result and clever way to link your analyses across scales
**Response 07:** Thank you for the positive comment.

L187 - can you discuss the potential for planktivore abundances to be over/under estimated in hyperabundant locations? How might this influence your results?

**Response 08:** Following your suggestion, we have expanded and split paragraph 3 of the discussion, which now raises this point in detail. It reads (lines 242-252):

“Underwater counts rely on visual fish detection, which has been highlighted as a potential source of methodological bias [e.g., 42–45]. Some planktivorous fishes, for example, have been found to have higher detectability than other reef fish groups [42], likely a consequence of their conspicuous nature and relatively high site association. Large schools of planktivores were a prominent feature of the hyper productive fish assemblages we highlight here, yet these involved both small, site-associated (e.g., damselfishes) and large and/or highly mobile groups (e.g., fusiliers). These highly mobile planktivores often feed and spend significant time away from the reef [e.g., 38,46], likely reducing, not increasing, their detectability, particularly where they are most abundant. Furthermore, field evidence indicates that larger school sizes per se do not increase fish detectability in visual counts [47]. Altogether, this suggests that the abundances recorded herein are conservative and that actual planktivore abundances may be considerably higher.”

L200 - can you discount the contribution of other pelagic subsidies to top predators here? It's not clear that planktivores are 'the' driving force, or one of several mechanisms by which pelagic subsidies raise fish biomass.

**Response 09:** We are not sure what your suggestion here is as this section exclusively refers to the high productivity of planktivores. It already disregards predators of planktivorous fishes or any other indirect pathways through which these subsidies may be incorporated within reef food webs. Although this distinction is emphasised in the Methods, following your next comment, it has been brought upfront.

L319 - this definition is key and should be noted in the main text and abstract to emphasise that spatial subsidies are bottom-up and planktonic. You do not consider spatial subsidies to other trophic levels

**Response 10:** This is a valid point and, again, we do agree that this definition could be more upfront. We have added a small excerpt in lines 67-69 to try and make it clearer from the beginning (also, see **Response 05**).

L323 - previous sentence says 'narrow definition', here it is 'coarse'. What is it?
**Response 11:** We have replaced ‘coarse’ with ‘narrow’, which indeed fits better here.

L324 - isn't predation on planktivorous fishes explicitly considered when you measure fish productivity, and this is why planktivore productivity corresponds with high overall biomass production?

**Response 12:** Not in the sense that the biomass production of piscivorous fishes that prey upon planktivores, for example, is not incorporated here, only that of their prey. Including the productivity of predators of planktivores (or any other indirect trophic links incorporating pelagic subsidies to reef food webs) would almost certainly increase our estimates (although more so in some locations than in others). This, therefore, makes our results, as they currently stand, conservative.

L338 - how minor? Can you give an estimate of the range of non-pelagic diet items in planktivorous reef fishes?
**Response 13:** Below we have listed a few instances where this has been quantified in the literature. This is by no means an exhaustive compilation of planktivorous fish diets, as this would be well beyond the scope of this manuscript. Yet, we felt it would be useful to exemplify some cases. From the 22 species of planktivorous reef fishes listed below, 10 had less than 5% of non-pelagic items (either by volume or proportional occupancy of grids, see studies), 13 had less than 10% and 16 had less than 20% non-pelagic items.

3.2% for *Chromis viridis* [*Chromis caerulea*, (Hobson & Chess, 1978)]

6-6.4% *Rhabdamia gracilis* (Hobson & Chess, 1978)

25% *Chromis agilis* (Hobson & Chess, 1978)

55% *Chromis margaritifer* (Hobson & Chess, 1978)

~2% *Abudefduf sexfasciatus* (Frédérich *et al.*, 2009)

~2% *Abudefduf sparoides* (Frédérich *et al.*, 2009)

~3% *Abudefduf vaigiensis* (Frédérich *et al.*, 2009)

0% *Chromis ternatensis* (Frédérich *et al.*, 2009)

42% *Dascyllus aruanus* (Frédérich *et al.*, 2009)

17% *Pomacentrus caeruleus* (Frédérich *et al.*, 2009)

0% *Naso hexacanthus* (Choat *et al.*, 2002)

46.5% *Naso annulatus* (Choat *et al.*, 2002)

8% *Thalassoma amblycephalum* (Huertas & Bellwood, 2020)

<1% *Cirrhilabrus exquisitus* (Huertas & Bellwood, 2020)

10% *Cirrhilabrus laboutei* (Huertas & Bellwood, 2020)

<1% *Cirrhilabrus lineatus* (Huertas & Bellwood, 2020)

1.5% *Cirrhilabrus punctatus* (Huertas & Bellwood, 2020)

5.8% *Cirrhilabrus scottorum* (Huertas & Bellwood, 2020)

2% *Pseudocoris yamashiroi* (Huertas & Bellwood, 2020)

42% *Ostorhinchus cyanosoma* (Frédérich *et al.*, 2017)

40.7% *Taeniamia fucata* (Frédérich *et al.*, 2017)

17.2% *Zoramia leptacantha* (Frédérich *et al.*, 2017)

We have now included more references to this statement and, to accommodate the 6 out of 22 species that had >20% non-pelagic items, we have slightly changed this sentence to (lines 375-377):

“Second, other sources of reef-based matter, such as fish or crustacean eggs often represent a minor to small proportion of the diet of diurnal planktivorous reef fishes (0 to 20% in most cases)”

L339 - presumably your surveys also do not census nocturnal planktivore abundances. Of course this still leads to an underestimate of planktivore productivity, but please note this limitation in the survey methods section too. If nocturnal planktivores are not in the abundance dataset, then it is also not relevant that these species have a higher degree of non-plankton food items…because your productivity estimates exclude all nocturnal species?

**Response 14:** Although you are correct in pointing out that our surveys, and also those from the Reef Life Survey dataset (which constitutes our ‘ocean-scale’ dataset), are made exclusively during the day, these surveys do include nocturnal fishes. This is because most nocturnal reef fishes take refuge on the reef during the day, whether in caves, crevices or simply resting above the substrate. Nocturnal planktivores are not an exception to this and have, been considered here as planktivores whenever detected and, as such, are included in the estimates presented.

L380 - what is the point cloud? Clearer to just say you fit a LOESS smoother to data to estimate relationship between relative trophic abundance and total abundance.

**Response 15:** This has now been changed as suggested. It now reads (lines 444-445):

“To estimate the relationship between the relative abundance of trophic groups, we fit a local polynomial regression smoother (LOESS). We used a near-unitary α parameter (>0.9) to minimise sensitivity to outliers.”

L462 - what do the PC axes represent about taxonomic diversity in this context? 

**Response 16:** PCoA Axis 1 was relatively highly correlated with taxonomic diversity (as represented by the average number of species per survey, Pearson’s *r* = 0.616), while Axis 2 was completely uncorrelated with taxonomic diversity (*r* = 0.01). Because both of these variables were highly correlated with planktivore abundance, they have been removed from the model structure before the fitting procedures. This information has now been explicitly incorporated in the Methods section, and we also mention all important a priori correlations between variables (line 561, lines 585-590).

L471 - what resolution are surface current pixels? Important because you are assuming some area is representative of plankton input to the reef site.

**AND**
L467-L474 - much more detail needed here. What units are the productivity and surface current metrics? What are the data underlying these layers? Are they time-averaged over seasons, years or decades? 
**Response 17:** Thank you for this suggestion. We have now detailed these procedures further, including the resolution of the datasets used to extract these variables and the time periods over which they were originally averaged. We hope this clarifies to what are these variables referring. It now reads (lines 572-583):

“Bathymetric slope is derived from the SRTM30_PLUS V6.0 [105,106], a 30 arc-second resolution, global digital elevation and seafloor topography model). Surface current speeds are derived from the ORAP5-ECMWF [107,108], a 1º resolution global ocean model driven by atmospheric forcing fluxes. Surface pelagic net primary productivity is obtained from PISCES-SEXTANT-IFREMER [108,109], a global 0.25º resolution biogeochemical model. Mean and maximum current velocities and pelagic net primary productivity are monthly averages for the period 2000–2014, and were interpolated and downscaled by Bio-Oracle [108], using a kriging model to achieve a final resolution of 5 arc-minutes. Bathymetric slope, mean and maximum current velocity values for each site were extracted from a buffering area with a radius of 10 km around each site, while surface pelagic net primary productivity was extracted from a buffering area with a radius of 30 km around each site. All extractions were accomplished using the *raster* package [103] in R.”

L492 - you are making model predictions, not forecasts (ie a time-series prediction)
**Response 18:** We agree that prediction is the most adequate term and have thus changed from ‘to forecast’ to ‘to predict’ as suggested, here and wherever else it had been used.

L501 - how many models were discarded? This is potentially problematic if certain metrics were difficult to fit, causing the bootstrapped posterior varies in size between each response metric.

**Response 19:** This information is now included, alongside model equations (see comments of Reviewer 2), for each model procedure in the sections *The trophic structure of Indo-Pacific reef fish assemblages and the productivity-biomass relationships of three key locations* and *Integrating datasets and predicting Indo-Pacific patterns of planktivore contribution to fish productivity*. Specifically, it can be found in lines 477-479, 490-492, and 600-601. Please, see **Response 01** for more details. Given that comparisons are always made within modelling procedures (i.e., between the ensemble posterior of the bootstrapped models), and never between modelling procedures, this should not be an issue.

L856-867 - This is useful oceanography context in the sup mat. Can you also consider how much intra-annual variability in your surface speed and primary productivity metrics? This is potentially important as UVC surveys are conducted at different times of year depending on location. I realise you cannot fully account for seasonal variability in these global UVC datasets, but some discussion of seasonality seems highly relevant for understanding planktonic spatial subsidies. More detail in these two remote sensing variables is also required in methods here, this will help others to build on your work.

**Response 20:** These variables are obtained as means of monthly averages from 2000-2014 from databases that summarise biogeochemical and oceanographic models. Therefore, accessing this level of detail is not possible at this time. We do agree, however, that variability in small-scale flows is likely key in driving spatial subsidies from the pelagic realm to reefs. Although this is only a hypothesis at this time, we nevertheless believe that satellite data will be too spatially-limited to further resolve these small-scale flows. This will require additional, long-term in-situ current speed data at varying depths and habitats on coral reefs, from which temporal and environmental variability can be resolved. We hope that this manuscript will stimulate some of this data to be collected and we are excited to see new developments in this field in the near future.


**Reviewer #2:
[identifies himself as Matthew McLean]**

PLOS Biology review:
Spatial subsidies drive sweet spots of marine biomass production

My overall opinion of this manuscript is positive, and I have very few criticisms. The authors did an excellent job in constructing this manuscript, which is thorough, detailed, and well written. As a reviewer, it is not often that I come across a paper this clean on the first submission, so bravo to the authors. Ultimately, this paper should make an important scientific contribution well suited for PLOS Biology.

**Authors:** Thank you for your insightful suggestions and your positive assessment of our manuscript. We are glad that you did enjoy it. We tried to incorporate as much as possible from your suggestions, and we are happy to provide responses to your comments below.

That said, I am not at all supportive of the terminology "sweet spots." I see what the authors are going for here - nice try - but this is going too far, trying to push a new flashy term with no actual meaning or value. Let's look at the actual definitions of both "sweet" and "sweet spot" and see if they capture the main ideas of this paper. From Google definitions:

Sweet. Adjective:
1) having the pleasant taste characteristic of sugar or honey; not salty, sour, or bitter. 
2) pleasing in general; delightful
3) (of a person or action) pleasant and kind or thoughtful
4) used for emphasis in various phrases and exclamations

I am going to argue that none of these definitions align with the main ideas of this paper.

Sweet spot. Noun:
1)  the point or area on a bat, club, or racket at which it makes most effective contact with the ball
2)  an optimum point or combination of factors or qualities

Number 1 is a definite no. Number 2 is leaning toward what the authors are referring to in their paper, but high planktivore biomass is not necessarily "an optimum point." So, I am going to argue that none of the available definitions of sweet nor sweet spot align with the main ideas of this paper. Moreover, let's look at antonyms of sweet. These include harsh, sour, bitter, disagreeable, nasty. So, if areas of high planktivore biomass are "sweet" then are areas of low planktivore biomass "sour" or "bitter"? And, by that nature, is Figure 4 showing us the sweet-to-sour planktivore index?

In all seriousness, simply replacing sweet spot by hot spot won't change anything about the paper or its quality and will be easily recognized and understood by any reader, so I highly encourage the authors to make this change.

**Response 21:** We appreciate your comments and have to admit that this is an aspect that we carefully considered before submission. We had a very vigorous discussion comparing the terms ‘hot-spot’ and ‘sweet-spot’. The main problem with hotspot is that the term comes with a lot of unhelpful baggage. The word hotspot has been used extensively in the biological literature and one of us has been involved in the ongoing scientific exchanges regarding the use and meaning of the term. The original definitions (*i.e.,* Myers *et al.*, 2000) were based on a combination of high biodiversity, high (plant) endemism and high local habitat degradation or threat. This was later applied to marine systems by (Roberts, 2002). It was then discovered that high endemism (and threat) did not coincide with high species richness, which was the primary focus of most studies (Hughes *et al.*, 2002). The working definition of marine hotspots was, therefore, subsequently restricted primarily to the description of an area of exceptionally high species richness. In the present study this was not the message we wanted to convey. We are not looking at richness but fish biomass and, more importantly, productivity – i.e. the ability of an area to supply fishable biomass. This is why we looked at the term ‘sweet-spot’.

As you note, the term ‘sweet-spot’ does not refer to anything sweet per se. However, it is not a ‘new flashy term’, but a rather old familiar (if colloquial) term in the fishing world. A quick search for the term ‘fishing sweet spot’ (i.e., on Google) results in a multitude of hits, from the “Sweet Spot Guide Service” on Facebook (with 3000 likes/followers) to a 2015 Fishing Magazine article on ‘The Sweet Spot’. In this context it is used to indicate a favourite, and productive, fishing location. It has even been used previously in fisheries science when describing model selection (cf. Collie *et al.*, 2016), Fish and Fisheries 17:101-125 “Ecosystem models for fisheries management: finding the sweet spot”). We use it in the former sense, i.e., to identify a potentially favourable fishing location. To address your concerns, and to avoid confusion, we highlight our definition early in the paper (lines 47-51), drawing attention to the meaning of the term in this particular application. It reads:

“Given their limitations (e.g., technology, economic, individual ability), fishers will actively search for the best fishing grounds, ‘sweet spots’ of fish concentration where effort or risks are minimised, and rewards are maximised [25,28]. We use the vernacular term ‘sweet spots’ to describe these areas of fish concentration where rewards are likely to be optimal (for a different use in the context of fisheries, see [29]).”

I would also encourage the authors to consider changing "spatial subsidies" to "planktivore subsidies" or "plankton subsidies" in the title because "spatial subsidies" is not very informative. The "spatial subsidy" referred to here is an influx of plankton into the system that is captured by planktivorous fishes, leading to exceptional biomass production. Saying "planktivore subsidies" in the title would make this immediately clear for readers.

**Response 22:** Thank you for the suggestion. While we do agree with the reviewer that the terms ‘planktivore subsidies’ and ‘plankton subsidies’ describe with a higher precision the nature of the process investigated, they are nevertheless an example of the broader term ‘spatial subsidies’. Here, we attempted to make the manuscript more general, and thus more interesting and useful for non-coral reef ecologists. While ‘plankton subsidies’ brings immediate information on the origin of these subsidies, it may not be readily recognisable as a type of ‘spatial subsidy’ by a readership unfamiliar with oceanographic concepts, and therefore we prefer to keep the latter term. We hope that this will stimulate research investigating whether increased biomass production from subsidies is a widespread phenomenon that can be found in other connected ecosystems. Nevertheless, please note that we now carefully ensure that we clarify what type of subsidies we are talking about many times throughout the manuscript, including in the Abstract (linking the terms ‘plankton subsidies’ [line 16] and ‘fish-mediated spatial subsidies’ [line 17]), in the Introduction (lines 43, 67-68, 74-75), Results (lines 160, 173, 178-179, 182), and in the Discussion (lines 241-242, 253-260, 270, 282).

My other main comment/question is why was abundance used to assess trophic structure instead of biomass, especially if biomass production is the main focus of the paper? For instance, Figure 2 shows trophic structure based on abundance. Planktivores, being small-bodied, schooling fishes, might show exceptionally high abundances, but not necessarily exceptionally high biomasses. Certainly, biomass is a better descriptor of trophic processes than pure abundance, given that trophic dynamics refers to the transfer of energy and material through the food web? It seems that these results could change substantially with biomass instead of abundance, with a much lower contribution from planktivores. The authors should explain and justify why abundance was used here instead of biomass, and I apologize if I am missing something obvious.

**Response 23:** Realising the importance of planktivores to the energetic processes highlighted here would almost certainly not have been possible had we focused on biomass from the start. As the reviewer correctly pointed out, due to their small body size, the contribution of planktivores to biomass would certainly be much smaller than their contribution to abundance. The disproportional influence of large-body sized individuals is an inherent aspect of biomass as a metric. As the reviewer mentioned, biomass is a good descriptor of trophic structure, as it has a direct link to ***storage*** of carbon and energy. However, because biomass in itself brings no information about the timeframe over which it was accumulated, biomass is not an adequate descriptor of ***trophic processes***. In this sense, a fish with 1 kg may have produced its body mass over a 2 month or a 20 year lifespan, yet these two cases have strikingly different implications for energetic dynamics. This information cannot be retrieved from biomass, severely limiting our ability to interpret ecosystem rates from it.

As an example, in a previous study (Brandl *et al.*, 2019) we have shown how small cryptobenthic reef fishes with negligible biomass contribute to important ecosystem processes through their quick lifespans, high mortality rates and, consequently, high biomass turnover rates. Many planktivorous fishes are halfway between the lifespan – and body size – of cryptobenthic fishes and larger reef fishes that dominate animal biomass. In another study (Morais & Bellwood, 2019), we have highlighted that the important contribution of planktivorous fishes to coral reef function would not be detected if we had only analysed their (minor) biomass. This pattern only became evident when productivity was analysed, as planktivores featured the highest discrepancy between productivity and biomass. While a number of studies investigated the spatial patterns of reef fish trophic biomass across the globe, few have highlighted planktivores as key contributors at this scale. Quantifying the real importance of small body-sized planktivorous reef fishes requires moving away from biomass and using different metrics.

While abundance may seem like a counter-intuitive choice of an indicator, as it is not directly linked to ecosystem structure or processes, it has at least one advantage over biomass. It does not a priori presumes differences between individuals. Here, each fish is treated as one, and therefore geographical patterns may emerge that could not be detected from an aggregate measure biased towards large body size like biomass. Indeed, as far as we know, it has never been shown before that planktivores dominate reef fish abundance wherever fish abundance is high, at least not in such a large spatial scale.

The workflow we follow here (1: establishing the numerical dominance of planktivores globally from their abundance; 2: finding a narrow association between this numeric dominance and overall high fish productivities; 3: concluding the role of these planktivores in driving extreme values of biomass production), in our opinion, has composed an intuitive narrative that can be particularlty interesting for non-specialists. This establishes important macroecological principles that may be useful elsewhere, also pointing to the previously overlooked role of planktivorous fishes in driving exceptional biomass production.

Line 18: what makes these locations "key"?
**Response 24:** These locations span a major biodiversity and abundance gradient, replicating the extensive differences in total fish abundance and planktivore proportion from the ocean-scale dataset, while providing a more geographically tractable dataset to be evaluated. We have now included “spanning a major biodiversity and abundance gradient” in this sentence (lines 14-15) to help clarify this issue.

Line 19: Figure 4 does not appear to show 2/3 of the global ocean. Do you mean 2/3 of the tropical ocean?
**Response 25:** This has been changed to “across the Indian and Pacific oceans” (lines 17):

Line 28: flows of energy and transport of matter in and of themselves are not spatial subsidies, so this first sentence is written incorrectly. The proper definition, however, is given in the following sentence. You should rewrite or combine these first two sentences to give a very clear and explicit definition of what a spatial subsidy is.
**Response 26:** We have rephrased these two sentences so that the relationship between ‘flows of energy and transport of matter’ and ‘spatial subsidies’ is less ambiguous. We hope it reads better (lines 26-29):

“Ecosystems are frequently connected by flows of energy and material [1–3]. These flows occur via the movement of resources (i.e., nutrients, detritus or prey) or consumers across ecosystem boundaries, characterising spatial subsidies [2,3]. Spatial subsidies involve at least one donor and one recipient ecosystem.”

Figure 1: great pictures! I'm jealous of Yen Yi Lee…

General comment: be consistent with the name Ha'apai, sometimes there is an apostrophe, sometimes not
**Response 27:** Thanks for noticing this. We have revised the manuscript and made sure the term is spelled consistently throughout.

Line 71: you chose 50% as the threshold to define hot spots, but why was this number chosen? This is glossed over very quickly, but its crucial to the entire paper. I can't seem to find the justification for this number anywhere in the manuscript.

**Response 28:** This was originally stated in lines 68-69 (“can underpin biomass production rates that outpace all other fish guilds combined”). This number was chosen because it sets the boundary between ***dominance***, which can be achieved at any value lower than this (only requiring that no other categories be higher) and the ***majority***, which implies that the sum of all other categories is unable to exceed the referred category (in this case, planktivores). We have now included the word ‘majority’ in this definition, which reads (line 76):

“(indicated by a majority [50% or more] of fish productivity comprised by planktivores)”

Line 79: you should specify whether this is proportion of species or abundances/individuals.
**Response 29:** This number refers to the proportion of individuals and has now been clarified (lines 83-84):

“This is evidenced by planktivores accounting for the largest proportion of fish abundance in most of the (…)”

Figure 2 panel B: the clear shift from invertivore-dominated to planktivore-dominated suggests a shift from benthic to pelagic energy channels, which is supported by the influence of slower currents and higher NPP. This might be worth mentioning in the discussion, particularly if a higher contribution of pelagic energy channels is key to supporting biomass hotspots. 

**Response 30:** We think it is risky to interpret these abundance data from an energetic perspective. Indeed this may be evidence of a switched energetic composition (i.e., among trophic groups), as we establish later after examining the biomass production data from the region scale dataset, and the predicted planktivore proportional productivity from the ocean scale dataset. However, we do not believe that this can be concluded at this point of the manuscript.

Line 123: HPD is for what interval/percentile?
**Response 31:** Thanks for pointing this lack. All HPD intervals used are 95% credible intervals. This has been clarified by including the percentile at first mention (line 109), and also in the Methods (line 424).

Line 126: "occupied a largely different area of the productivity spectrum" is really vague, sounds overly technical, and is not very meaningful. You should rewrite this in a more precise manner to say exactly what the difference is (i.e., raja ampat is higher in both total productivity and planktivore productivity).
**Response 32:** This paragraph has been rewritten because of changes in data analyses (correlations instead of regressions, see **Reponse 03** to Reviewer 1) and this sentence changed as suggested (lines 134-135).

Line 146: I am not really sure what you mean here by "reconciling the energetic implications" try to use less ambiguous and more precise wording to help the reader follow and understand the flow of the storyline.
**Response 33:** This has now been edited for clarity. It now reads (lines 160-162):

‘To assess the geographic extent of planktivore-mediated subsidies, we explored the energetic implications of high planktivore productivity (revealed by the analysis of these three focal locations) within the ocean-scale dataset.’

Line 151: maybe I am not understanding what you are saying here, but if all other trophic groups remain static and planktivore abundance goes up, isn't it obligatory that planktivores would account for more of the productivity, i.e., relative planktivore productivity would increase? If I am missing the idea of the sentence, please try to clarify.

**Response 34:** This sentence solely describes the procedure used to model the proportional productivity of planktivores. We hypothesised that, given that higher fish abundances were associated with higher planktivore proportional abundances, as planktivore abundance increased, their proportional productivity would also increase. This modelling procedure was key, in the following section, to be able to predict proportional planktivore productivity for the ocean-scale dataset. It is uncertain how this section of the results could be further clarified, but we hope that the change in the topic sentence (above, **Response 33**) has helped to clarify the meaning of this paragraph.

Line 154: "would result in more than 50% of total productivity based on planktivores" I think you can rewrite this more clearly, something like "more than 50% of total productivity would be accounted for by planktivores"

**Response 35:** Changed as suggested (lines 168-169).

Line 159: the phrase "planktivore subsidies" here immediately makes me think that the title would be more accurate and easier to interpret if it said "planktivore subsidies" or "plankton subsidies" rather than just "spatial subsidies" which by itself is not very informative.

**Response 36:** We do agree with the reviewer that ‘spatial subsidies’ does not clearly incorporate any specific information on the nature of these subsidies. However, please see our **Response 22** for the reasoning why we think that keeping ‘spatial subsidies’, instead of ‘plankton subsidies’ or ‘planktivore subsidies’ is the best option for this manuscript in particular.

Line 165: what's the definition of "pelagic subsidy" here? Is this identical to "planktivore subsidy" or is it distinct? If they are interchangeable, it might be better to stick with one term only for consistency.

**Response 37:** The two terms are used interchangeably, and a clear definition of the two is given in the Methods (lines 318 to 321 of the original submission). We have, nevertheless, revised the usage of the term ‘pelagic subsidies’ throughout the manuscript. The two instances where it occurred have been replaced with ‘spatial subsidies’ (line 67) and ‘plankton subsidies’ (line 179), respectively. However, we have kept both ‘plankton subsidies’ and ‘pelagic subsidies’ in the Methods section where the definition can be found, as this is critical to establish the equivalence and because many studies refer to the term ‘pelagic subsidies’.

Line 177: if these predictions were made under a Bayesian framework, why do you present means and standard deviations here instead of medians and credible intervals/HDP like elsewhere in the paper?

**Response 38:** Because the predictions from the Bayesian modelling framework are done at the site level. These predictions encompass the small dots of this figure: each dot represents the predicted proportional planktivore productivity of a unique site. The means and standard deviations here were exclusively used to summarise these site-level predictions at the ecoregion level, so that visualising patterns became easier: no inferences are made based on them.

Line 179: is the 20% expected value a model estimate, or is this just saying that 100% divided equally by 5 trophic groups would yield 20% each? If the latter, perhaps this should be rethought because in reality, one would never expect equal representation across trophic groups.

**Response 39:** Your suggestions are both correct. On a completely uninformative context, assuming that trophic groups are absolutely independent from one another (i.e., that there is no overlap in resource usage between any of the trophic groups), without awareness of asymmetries in other features (such as morphological features or body size) between trophic groups, one could hypothesise that any ecosystem processes on which fishes take part could be equally divided among trophic groups. We agree with the reviewer that this is not a realistic model of trophic structure on coral reefs, but we suspect the reason why we agree is because we share much of the prior knowledge about the subject. For uninformed readers, this ‘parity hypothesis’ may be acceptable and, therefore, we keep it merely as a reference point with which to contrast the modelled proportion of the productivity composed by planktivores.

Line 186: perhaps you should say "in almost any region" instead of "almost anywhere" to make the distinction between regions and sites. For instance, you show that these hot spots can arise in any region, but they aren't necessarily possible on any site within a region since they arise under certain environmental conditions.

**Response 40:** Thank you, we have changed as suggested (line 201).

Line 189: somewhere in the manuscript it might be good to really stress the idea that as you add more and more fish to a system, it's basically just more and more planktivores. It might also be worth thinking about/discussing whether other trophic groups hit a ceiling, whereas the biomass ceiling for planktivores is much less limited.
**Response 41:** We agree with the reviewer that these topics are key for our manuscript. Therefore they have been discussed in detail (although in terms of productivity, which we felt is more adequate than biomass given the focus of the manuscrupt) in the third paragraph of the Discussion (lines 218-240) and, particularly, in the supplementary material, section ‘*Spatial constraints of fish productivity on coral reefs*’.

Line 196: you haven't really touched on external productivity yet in the manuscript, it would be good to make this clear for the reader that you are referring to an influx of plankton, particularly in areas with slow currents and high NPP.
**Response 42:** Thank you for the suggestion. We have clarified this by changing this sentence to ‘Importantly, by vectoring external productivity in the form of plankton(…)’ (lines 218-219).

Line 215: you haven't spoken about nutrition yet in the paper, so what do you mean specifically here? Yes, greater fish biomass leads to greater energy flow/production, but if, for instance, planktivores are low in nutrient concentration, then there isn't really an expansion of the "nutritional footprint." Moreover, I would encourage you to use wording that is a bit more meaningful and less ambiguous than "energetic and nutritional footprint"
**Response 43:** The word ‘nutritional’ was a misnomer, as it should have been ‘nutrient’ instead. Thank you for pointing this out, it has now been corrected (lines 240).

Line 380: "the point cloud" hasn't been defined yet, so you might want to give a more specific description of what you are referring to here (i.e., data were first plotted and then fitted)
**Response 44:** We have suppressed any references to the ‘point cloud’, as described in **Response 15** to Reviewer 1. This has now been changed to (lines 444-445):

“To estimate the relationship between the relative abundance of trophic groups, we fit a local polynomial regression smoother (LOESS). We used a near-unitary α parameter (>0.9) to minimise sensitivity to outliers.”

Line 390: what were these striking distinctions?
**Response 45:** We complemented this sentence with further information. It now reads (lines 455-457):

“This unveiled striking distinctions in the relative status of abundant species between the locations. These patterns were then further explored by contrasting total fish abundance, α, β and γ diversities, size and trophic structure, and productivity-biomass relationships between locations”

Paragraph beginning on line 398: I would encourage you to provide the equations for the Bayesian models you performed

Moreover, because model equations are not provided, it is not clear to me at what scale each of the models were performed? Were the replicates transects, sites, or regions? Were the models nested with random effects? This needs to be specified. Additionally, it should be specified at which spatial scale the predictor variables are measured and enter these models. 
**Response 46:** Thank you for this suggestion, which we agree will aid in making the analyses clearer. We have now considerably expanded our description of the modelling procedures, specifying the scales at which predictors have been measured and entered in the model, as well as the model equations including distributions, coefficients estimated, and priors used. The new details have been added in multiple excerpts in the Methods sections *The trophic structure of Indo-Pacific reef fish assemblages and the productivity-biomass relationships of three key locations* and *Integrating datasets and predicting Indo-Pacific patterns of planktivore contribution to fish productivity*. We feel that these additional details have indeed made our methods clearer and easier to reproduce. Thank you again for this suggestion.

Line 422: here, again I am not sure what "trophic imprint" refers to, so you would be better off clearly stating the result you saw.
**Response 47:** This has been rephrased into “This revealed a clear separation in the dots, particularly between those of Raja Ampat and the other two localities.” (lines 498-499), which we feel is much clearer. Thank you for the suggestion.

Line 441: here, I initially saw "after using model selection…" and thought that not nearly enough detail was provided on how model selection was performed. I then found that the subsequent paragraphs explain this procedure. I suggest rewriting this to make this very clear. For instance, something like, "We then performed model selection (described below) to narrow down a final set of variables, which were then used to predict the likely contribution of planktivores…"
**Response 48:** Thank you for your comment. We agree that your suggestion reads better and have, therefore, changed accordingly (lines 538-540).

Line 499: do you mean number of effective samples?

**Response 49:** Yes, effective sample sizes. This mistake has been amended (line 434), thanks for spotting it.

Line 812: the second and third "comprising" should be changed to "comprised"
**Response 50:** This has been changed as suggested, thank you.

Line 879: this should be "Assuming that 40-70% of this is consumed, ~50% of this being by herbivorous fishes…" Also, what is the basis for assuming 40-70% consumption?
**Response 51:** This has been changed as suggested (lines 1139-1142). The 40-70% range of values (alongside the ~50%) was sourced as a back of the envelope average proportion of turf production consumed by herbivores from the references cited. To make this clearer, we also included the note “(average turf production consumption rates from(…)”. We hope that this has made the origin of these proportions clearer.

Line 880: to avoid confusion with arithmetic mean, change this to "this would result in a maximum expected…"
**Response 52:** Thank you for spotting this potential confusion, we have changed as suggested (line 1141).

Line 894: I encourage you to use a much simpler word than "impinges"
**Response 53:** The term “impinges” is common in the oceanographic literature to describe how water masses interact with geomorphologic features. We have nevertheless replaced it with ‘reaches’ as suggested (line 1155).

Line 909: what is the "energetic matrix"? Try to be very clear about what you mean here
**Response 54:** We have rephrased this sentence with (lines 1169-1171):

“(…) planktivorous fishes considerably expand the resource pool, and therefore the energetic and nutrient footprint, of coral reef consumers.”

Line 915: you need to state that you compared these things between the two datasets, otherwise it reads as if you just compared distributions of the four variables to each other
**Response 55:** Thank you for the suggestion. We have now explicitly clarified this by including ‘between datasets’ after ‘the four final predictors of the model’ (line 1219).

Figure S1: Haapai is cut off the map
**Response 56:** This has now been adjusted.

I am signing my review for transparency and invite the authors to contact me if they have any questions.

Sincerely,
Matthew McLean
**Authors**: Once more, thank you for your detailed conceptual evaluation of our manuscript and positive comments, which we deeply appreciate.

**Reviewer #3:**

This paper explores whether energetic subsidies from plankton-rich waters around some coral reefs are the basis for high fish abundance and productivity in those areas - and whether consequently, planktivores as a trophic group are the key drivers of high productivity on coral reefs. This is a really interesting paper with a conclusion that could be vital to coral reef management. The authors make excellent use of a valuable global dataset and the manuscript is well-written with really excellent figures.

**Authors:** Thank you very much for your positive comments and for raising very relevant points regarding our hypothesis tests. To address your concerns, we have now performed two additional analyses, which we describe and provide the results below.

My main concern with the paper surrounds the potential impact of current and historical fishing on the data, and on the conclusions drawn. At no point in the manuscript is the effect of fishing on the observed abundance, productivity, size structure or trophic structure of the communities discussed, and yet fishing is likely to alter all of these metrics. Furthermore, the fishing pressure across the sites is likely to vary significantly at all of the spatial scales considered. To my mind, without accounting for fishing effects, it is very hard to draw conclusions about differences in trophic structure and dominant trophic groups between sites. For example, under high fishing pressure you would expect a decline in large carnivores, falling into the GC category - but with their removal, you might also expect an increase in both the abundance and the relative contribution to the community of lower trophic levels (their prey) - including small planktivores that contribute to the conclusions drawn in this study. It's also interesting to note from Figure 2 that arguably the most remote sites in terms of fishing pressure, in the middle of the Pacific, also seem to show a trend for reduced dominance of planktivores. Indeed, they appear to have a greater proportion of omnivores, herbivores and carnivores in general - which you might interpret as a more in-tact coral reef trophic structure. I think this is also particularly important because the sites with the greatest overall abundance in the coral triangle, are data points that make a disproportionally important contribution to the overall conclusion of this study - the very high productivity sites for example, are found here. This area is also arguably much more heavily fished, in general than locations in Australia or the central Pacific. Perhaps the authors have thought about this and selected data from sites with minimal / no fishing, in which case this justification needs to be clear. Otherwise, some metric of fishing pressure ought to be included in the analyses to quantify its contribution to the observed patterns in the data.

**Response 57:** This is a relevant and fair point, thanks for raising it. As mentioned, we strived to only incorporate sites that had limited to no fishing. However, we obviously cannot guarantee that this is the case for all sites. Therefore, following your suggestion, we have devised two additional analyses to address your concerns. As you will see, these analyses provide evidence that fishing is not an important factor at play here – at least not one that could explain our results. This is mainly because:

**1) We found no evidence of an effect of a proxy for fishing (‘gravity of human impacts’, Cinner et al. 2018 PNAS) on either fish biomass or productivity, between or within localities.** This metric of human impacts has a foundation in human behaviour, and has been found to outperform other proxies of human impacts, such as human population size or travel time to markets alone (Cinner *et al.*, 2018). Although there is some variability in human gravity among sites in two of our localities (Raja Ampat and Ha’apai, see new Fig. S6), the maximum value across any of our sites is only 5.5 (Fig. S6A), around 38,000 times lower than the maximum human gravity that coral reefs are exposed to, according with (Cinner *et al.*, 2018). This supports our hypothesis that none of our sites are exposed to intense fishing activities. Furthermore, we found no relationship between gravity (predictor) and biomass or productivity (responses), as evidenced by the flat lines in Figs. S6B and S6C). Indeed, all models including gravity performed worse than a model that only included different intercepts for the different localities (Supplementary Text, section *Testing for the effects of human impacts*). Not surprisingly, including human gravity as one of the predictors of relative planktivore productivity in our predictive model (*i.e.,* from Figs. S5 and S7, used to predict for Fig. 4) resulted in this variable being eliminated from the model due to lack of explanatory power (Methods, lines 548-549, 567-569, 592-600).

**2) We found no evidence that trophic release of planktivores due to the removal of predatory fishes by fishing was operating in our selected locations.** Support for this alternative hypothesis would come in the form of a negative relationship between the productivity of predatory fishes and planktivorous fishes. Indeed, contrary to this expectation, we found a positive relationship between the productivity of predators and planktivores in all three locations (Fig. S6D). Again, this is strong evidence for a lack of top down effects from fishing on planktivores via predatory fishes, instead providing support for our overall hypothesis that intensified planktivore productivity (which we attribute to local scale oceanographic processes) is passed on to their predators. As a result, places with high planktivore productivity also tend to have high predatory fish productivity.

Following these analyses, now provided in the Suppplementary Text, section *Testing for the effects of human impacts* and Fig. S6, we are very confident that our results represent genuine effects of pelagic subsidies, via planktivorous reef fishes, increasing the overall productivity of reef fish assemblages. We also incorporate these lines of evidence in the Results section (lines 127-129) and in the Discussion (lines 210-217). We thank you once more for your suggestion, since we believe that this analysis has made the manuscript stronger.

Also on fishing, I think it is important to include some information on the target species in these fisheries. The key message of the paper is that energy subsidies support "sweet spots" for reef fisheries but without qualifying whether the planktivores that make up this high productivity are targeted, or indeed available to fishermen on these reefs, the message is a little strong. I can fully believe that reefs in locations with high water flow or upwelling support abundant planktivore communities - but unless these fish are targeted, or contribute significantly to energy flow up the food chain to groups that are targeted, then I wonder if they are not really a separate ecosystem - at least from the perspective of the fishermen. Perhaps more information could be pulled into the main paper about the identities of the species contributing to the overall patterns - and the extent to which these are targeted - or consumed by predators that are.

**Response 58:** This is a valid point, and we do agree with the reviewer that this extra productivity will only become useful for humans if it constitutes target species or prey of target species. We had originally alluded to this in the Introduction and Discussion. Examples include baitfish, fusilier and unicornfish fisheries, which are important fisheries in several places in the Indo-Pacific, including Indonesia, Solomon Islands and the Philippines (see Alcala & Gomez, 1985; Bellwood, 1988; Dalzell et al., 1996; Pet-Soede et al., 2001; Campbell & Pardede, 2006; Humphries et al., 2019). We agree with the reviewer that this would compose an interesting additional component. However, we believe that exploring this aspect with the depth it deserves will require an entirely new manuscript with scope much more applied than this one. As a compromise, we have substantially expanded our reference base to provide further support for this idea (lines 261-281, particularly lines 271 and 275), also including a sentence highlighting our finding of a positive relationship between the productivity of planktivores and that of predatory fishes (Fig. S6 now, see **Response 57** above). We intend on exploring the importance of planktivorous reef fishes for coral reef fisheries in a follow-up study.

I really like this paper, and do not fundamentally disagree with the conclusion, but I think this is a gap that really needs addressing before publication. I would also like to acknowledge that I am not well-versed in the Bayesian analyses employed here - and whilst to my understanding these analyses seem thorough and appropriate, it will be important to have the review of someone with more expertise to cover that part of the paper. Below I include some more minor comments to assist with re-working of the paper.

**Response 59:** We appreciate your enthusiasm with our paper. As you may see above, Reviewer 1 had raised a few points related to the Bayesian modelling procedures, which we were happy to clarify. Please, refer to **Responses 01**, **02** and **03** for this.

Introduction

Line 28: the first line suggests that flows of energy and transport of matter are a definition of spatial subsidies - I think this is misleading.
**Response 60:** This and the next sentence have been rephrased following a comment by Reviewer 2, which was similar to yours. The rephrased sentences now read as (lines 26-28):

“Ecosystems are frequently connected by flows of energy and material [1–3]. These flows occur via the movement of resources (i.e., nutrients, detritus or prey) or consumers across ecosystem boundaries, characterising spatial subsidies [2,3]. Spatial subsidies involve at least one donor and one recipient ecosystem.”

Figure 1 - the reference to this figure, and the legend that goes with it, I do not think is appropriate - they are nice images that convey the idea of concentrated fishing effort - and planktivore-dominated reef fish assemblages - but they don't provide evidence that fishers do not fish randomly - and panel B definitely doesn't show how plankton underpins sweet spots of fish productivity - it just shows aggregations of planktivores on a reef.
**Response 61:** Thank you for your thoughts on this. While we do agree with your interpretation of the meaning of each photo separately, please bear in mind that the concepts illustrated with these photos are complex, and thus hard to capture in a single or even a set of images. These images represent two critical features associated with the paper, which you defined well: the first shows concentrated fishing effort, the second concentrated abundance/biomass [also presumably productivity] of planktivorous reef fishes. We do not have access to a set of photos that more readily presents the concept of ‘selection of disproportionally productive habitats by fisherfolk’ (and we are not even certain that one such exists). The combination of these two photos was meant to illustrate this concept with as much accuracy as possible. With that being said, we have changed the title of the caption from “***Fishers do not fish at random***” to “***Neither fishers nor fishes are randomly distributed across reef scapes***”. We hope that the reviewer finds this more adequate.

Line 64: dissecting is a strange choice of word - examining / disentangling perhaps?
**Response 62:** Thank you for the suggestion, however, we prefer to keep the term dissecting, as it implies a thorough, focused and deep examination.

Results

Lines 105 - 108: I find the reporting of the data confusing here - e.g. 60% and 206% higher in Raja Ampat compared with Lizard and Ha'apai - it's an effort to work out which numbers relate to which comparison - it would be clearer to be explicit about each.
**Response 63:** This has been rephrased for clarity [please, note that some coefficients have suffered minor numerical changes as another round of 1,000 bootstrapped analyses were run]. It now reads (lines 111-113):

“Differences in standing biomass between locations were less extreme, being 55% higher in Raja Ampat (4.15 t ha^-1^) compared with Lizard Island (2.68 t ha^-1^, *β*_bio_ = 1.55, HPD = 0.94 to 2.34) and 199% higher in Raja Ampat than Ha’apai (1.39 t ha^-1^, *β*_bio_ = 2.99, HPD = 1.98 to 4.29).”

Line 125 - I don't think that Figure 3D shows how carnivores comprise a fraction of the productivity of planktivores - perhaps this is supposed to point to a different panel?

**Response 64:** Thank you for spotting this: it should indeed have been referring to panel 3E. This has now been corrected.

Discussion

Line 187 - how are you defining over-representation? If it's over-represented you must be comparing it to some expectation of what it's representation should be?
**Response 65:** This refers to the fact that planktivores frequently dominate the abundance (>50%) at high-fish abundance sites, often comprising 80% or more of the individuals in the sites with the highest abundances. Nevertheless, we think the reviewer has a point here, and thus we have replaced “are over-represented in” with “dominate”, which should bring less unintended confusion (line 202).

Line 215 - Another thing that struck me was the assumption that the reef-planktivores captured by your surveys were obtaining their food from large areas - depending on the identity of these fish species, many are site-attached to reefs - relying on them for structure for example - and so not moving significant distances. Any those that are moving are arguably not part of the reef, or the reef fishery. Perhaps you only mean that their food comes from a larger area, and is transported to them, in their location on the reef - but this needs to be clear.

**Response 66:** Yes, you are correct in that we refer to the fact that their food comes from a larger area, largely transcending the boundaries of the reef. To clarify this, we have included “carried by ocean currents” between “productivity” and “from larger areas” (line 239) in this section, and also included “(transported to the reef by ocean currents)” in a related sentence present in the section *Spatial constraints of fish productivity on coral reefs*, where we expand on this idea.

Supplementary material

Line 949-951 - I think it's really interesting here that you are finding "sweet spots" in areas where you don't expect them to occur due to high planktonic productivity - 20% is not a small amount of variation and so digging into what is happening in these systems would be really interesting. Detrital recycling is of course the other process by which high productivity is achieved on reefs with limited primary production. Would the dataset allow you to explore the role of that in your observed patterns?
**Response 67:** Thank you for your comments. We agree that this is a very interesting observation and, in our opinion, this may hold the secret to understand smaller-scale ‘sweet spots’ of biomass production (i.e., like those observed among counts within the region-scale dataset). While we were positively surprised with the 80% of explanation achieved (a number quite rare in this type of study), we feel like we have exhausted the variables with the potential to add information to this model in the current dataset. We believe that further knowledge will rely on finer-scale, in-situ water movement and plankton transport data that cannot be retrieved or approximated by satellite applications (or any remote sensing data).

Figure S4 - I may be misunderstanding the analyses here but I don't understand the fit drawn through the data in the Haapai panel - why is the line so shallow and the points much steeper?

**Response 68:** We believe that this was a visual effect from scaling the dots by the relative productivity of planktivores. Following a comment from Reviewer 1 (see **Response 06**), this analysis was redone as a correlation and its results combined, and can now be seen as Fig. S3. All dots are now scaled to a common size, leaving the relationship between the trendlines (Pearson correlations) and data points easier to perceive.

**References cited:**

Alcala, A.C. & Gomez, E. (1985) *Fish yields of coral reefs in Central Philippines*. pp. 75–81.

Bellwood, D.R. (1988) Seasonal changes in the size and composition of the fish yield from reefs around Apo Island, Central Philippines, with notes on methods of yield estimation. *Journal of Fish Biology*, **32**, 881–893.

Brandl, S.J., Tornabene, L., Goatley, C.H.R., Casey, J.M., Morais, R.A., Côté, I.M., Baldwin, C.C., Parravicini, V., Schiettekatte, N.M.D. & Bellwood, D.R. (2019) Demographic dynamics of the smallest marine vertebrates fuel coral reef ecosystem functioning. *Science*, **364**, 1189–1192.

Campbell, S.J. & Pardede, S.T. (2006) Reef fish structure and cascading effects in response to artisanal fishing pressure. *Fisheries Research*, **79**, 75–83.

Choat, J.H., Clements, K.D. & Robbins, W.D. (2002) The trophic status of herbivorous fishes on coral reefs. *Marine Biology*, **140**, 613–623.

Cinner, J.E., Maire, E., Huchery, C., MacNeil, M.A., Graham, N.A.J., Mora, C., McClanahan, T.R., Barnes, M.L., Kittinger, J.N., Hicks, C.C., D’Agata, S., Hoey, A.S., Gurney, G.G., Feary, D.A., Williams, I.D., Kulbicki, M., Vigliola, L., Wantiez, L., Edgar, G.J., Stuart-Smith, R.D., Sandin, S.A., Green, A., Hardt, M.J., Beger, M., Friedlander, A.M., Wilson, S.K., Brokovich, E., Brooks, A.J., Cruz-Motta, J.J., Booth, D.J., Chabanet, P., Gough, C., Tupper, M., Ferse, S.C.A., Sumaila, U.R., Pardede, S. & Mouillot, D. (2018) Gravity of human impacts mediates coral reef conservation gains. *Proceedings of the National Academy of Sciences*, **115**, E6116–E6125.

Collie, J.S., Botsford, L.W., Hastings, A., Kaplan, I.C., Largier, J.L., Livingston, P.A., Plagányi, É., Rose, K.A., Wells, B.K. & Werner, F.E. (2016) Ecosystem models for fisheries management: finding the sweet spot. *Fish and Fisheries*, **17**, 101–125.

Dalzell, P., Adams, T.J.H. & Polunin, N.V.C. (1996) *Coastal fisheries in the Pacific Islands*. *Oceanography and Marine Biology: an Annual Review* (ed. by A.D. Ansell), R.N. Gibson), and M. Barnes), pp. 395–531.

Frédérich, B., Fabri, G., Lepoint, G., Vandewalle, P. & Parmentier, E. (2009) Trophic niches of thirteen damselfishes (Pomacentridae) at the Grand Récif of Toliara, Madagascar. *Ichthyological Research*, **56**, 10–17.

Frédérich, B., Michel, L.N., Zaeytydt, E., Bolaya, R.L., Lavitra, T., Parmentier, E. & Lepoint, G. (2017) Comparative Feeding Ecology of Cardinalfishes (Apogonidae) at Toliara Reef, Madagascar. *Zoological Studies*, 14.

Hobson, E.S. & Chess, J.R. (1978) Trophic relationships among fishes and plankton in the lagoon at Enewetak atoll, Marshall Islands. *Fishery Bulletin*, **76**, 133–153.

Huertas, V. & Bellwood, D.R. (2020) Trophic separation in planktivorous reef fishes: a new role for mucus? *Oecologia*, **192**, 813–822.

Hughes, T.P., Bellwood, D.R. & Connolly, S.R. (2002) Biodiversity hotspots, centres of endemicity, and the conservation of coral reefs: Biodiversity of coral reefs. *Ecology Letters*, **5**, 775–784.

Humphries, A.T., Gorospe, K.D., Carvalho, P.G., Yulianto, I., Kartawijaya, T. & Campbell, S.J. (2019) Catch Composition and Selectivity of Fishing Gears in a Multi-Species Indonesian Coral Reef Fishery. *Frontiers in Marine Science*, **6**, 378.

Loreau, M., Mouquet, N. & Holt, R.D. (2003) Meta-ecosystems: a theoretical framework for a spatial ecosystem ecology. *Ecology Letters*, **6**, 673–679.

Morais, R.A. & Bellwood, D.R. (2019) Pelagic Subsidies Underpin Fish Productivity on a Degraded Coral Reef. *Current Biology*, **29**, 1521-1527.e6.

Myers, N., Mittermeier, R.A., Mittermeier, C.G., da Fonseca, G.A.B. & Kent, J. (2000) Biodiversity hotspots for conservation priorities. *Nature*, **403**, 853–858.

Pet-Soede, C., Van Densen, W.L.T., Pet, J.S. & Machiels, M.A.M. (2001) Impact of Indonesian coral reef fisheries on fish community structure and the resultant catch composition. *Fisheries Research*, **51**, 35–51.

Polis, G.A., Anderson, W.B. & Holt, R.D. (1997) Toward an Integration of Landscape and Food Web Ecology: The Dynamics of Spatially Subsidized Food Webs. *Annual Review of Ecology and Systematics*, **2**, 289–316.

Polis, G.A. & Hurd, S.D. (1996) *Allochthonous Input Across Habitats, Subsidized Consumers, and Apparent Trophic Cascades: Examples from the Ocean-Land Interface*. *Food Webs* (ed. by G.A. Polis) and K.O. Winemiller), pp. 275–285. Springer US, Boston, MA.

Roberts, C.M. (2002) Marine Biodiversity Hotspots and Conservation Priorities for Tropical Reefs. *Science*, **295**, 1280–1284.
