# Supplementary material for: Spatial subsidies drive sweet spots of tropical marine biomass production
Source: PLoS Biol. 2021 Nov 2;19(11):e3001435. doi: 10.1371/journal.pbio.3001435 (PMC8562822; doi:10.1371/journal.pbio.3001435)
Supplement: Attachment — Submitted filename: Morais et al. Responses to Reviewers.docx [file pbio.3001435.s013.docx]

**REVIEWERS' COMMENTS:
Reviewer #1:
[identifies himself as James Robinson]**

The authors have been exceptionally thorough in dealing with my statistical concerns, and I appreciate the detailed responses and extra analyses now in the manuscript. The revised methods demonstrate the reasoning and robustness of the bootstrapped Bayesian approach (useful to see the comparison of HPDs in Table 1, thanks). The trophic productivity models are also now improved, and I think the new analyses of human gravity and piscivore productivity add great context.

Just a last thought regarding the overall implication that most reef fisheries benefit from planktonic sweet spots (L279-286), and the comment from R3 on target species. As you write, high biomass production will support specific fisheries - gears with selectivity for planktivores (nets) or consumers in higher trophic levels (handlines). But nets and traps also target lower trophic level species, like herbivores, which likely correlate with benthic productivity but not plankton sweetspots. Perhaps 'herbivore sweetspots' would be predicted by a different set of variables (algal cover, depth, coral cover, rugosity, L1137) that exist in different sites to plankton sweet spots (though are constrained by algal productivity). Regions with both sweetspots would be complementary - and perhaps best sustain diverse reef fisheries by offering resilience against ecosystem change (a portfolio effect). Even though herbivore productivity is far smaller than planktivore (L1134), herbivores
dominate catch on many heavily fished reefs (Kenya, see Hicks & McClanahan 2011; Seychelles, my work with Nick Graham; Indonesia, Humphries et al. 2019). 

This idea is beyond the scope of your study. But you could note in the discussion that some key reef fisheries (herbivores) are unlikely to benefit/correlate with plankton sweetspots. I think this is important for considering how trophic group productivity will influence fisheries on degraded reefs (L290), e.g. in Morais et al. 2020 (Func. Ecol.) you showed that herbivores drive biomass and productivity increases on turf-dominated reefs.

The final figures are great, as usual from this team. One minor suggestion for Figure 3B, 3C + S2B,C - adding jitter/dodge to points would reveal error bars.

The study is a big advance for reef energetics + fisheries - I recommend acceptance and look forward to reading in print.

**Response**: First and foremost, thank you for your constructive criticism of our manuscript throughout the reviewing process. We are happy that the changes we made have solved the concerns you had initially.

In response to your second suggestion, i.e., to reveal error bars in Figs 3B and 3C and S2B and S2C, we have now dodged the points and segment lines, making the locations individually recognisable.

In response to your first suggestion, i.e., to consider that herbivore-focused fisheries are unlikely to benefit from planktivore sweet spots, we have now included an additional paragraph in the Discussion. This paragraph expands on your ideas and incorporates a couple of sentences originally in the second last paragraph of the Discussion. In this new paragraph, we recognise that not all fisheries will benefit from planktivorous fish-mediated subsidies, citing some of the references you suggested. Furthermore, we also bring forward your proposition that herbivorous and planktivorous fish production are likely to have a complementary role in determining sweet spots of fisheries catches.

This paragraph reads as:

“Some tropical fisheries are unlikely to benefit, at least directly, from the plankton-subsidies we recognise here. Trap and seine net fisheries, for example, are frequently dominated by herbivorous fishes (e.g., [64–66]), which tend to respond to habitat and/or benthic processes [65,67,68]. However, given their intrinsically strong relationship with total fish productivity, planktivore sweet spots are likely to be disproportionally important for many reef-based fisheries [35–38]. Nevertheless, it is conceivable that seascapes which feature favourable conditions for both plankton-subsidy and herbivore sweet spots will offer maximised yield opportunities for tropical reef fisheries.”

We believe that this has encapsulated your reasoning above. Thank you once more for the suggestions.
